# Investor sentiments and stock markets during the COVID-19 pandemic

**DOI:** 10.1186/s40854-022-00375-0

**Published:** 2022-07-05

**Authors:** Emre Cevik, Buket Kirci Altinkeski, Emrah Ismail Cevik, Sel Dibooglu

**Affiliations:** 1 Tekirdag Namik Kemal Universitesi, Tekirdaǧ, Turkey; 2 Kırklareli University, Kırklareli, Turkey; 3 University of Sharjah, Sharjah, United Arab Emirates

**Keywords:** COVID-19, Investor sentiment, Stock market returns, Volatility, G12, G14, C22, C23

## Abstract

This study examines the relationship between positive and negative investor sentiments and stock market returns and volatility in Group of 20 countries using various methods, including panel regression with fixed effects, panel quantile regressions, a panel vector autoregression (PVAR) model, and country-specific regressions. We proxy for negative and positive investor sentiments using the Google Search Volume Index for terms related to the coronavirus disease (COVID-19) and COVID-19 vaccine, respectively. Using weekly data from March 2020 to May 2021, we document significant relationships between positive and negative investor sentiments and stock market returns and volatility. Specifically, an increase in positive investor sentiment leads to an increase in stock returns while negative investor sentiment decreases stock returns at lower quantiles. The effect of investor sentiment on volatility is consistent across the distribution: negative sentiment increases volatility, whereas positive sentiment reduces volatility. These results are robust as they are corroborated by Granger causality tests and a PVAR model. The findings may have portfolio implications as they indicate that proxies for positive and negative investor sentiments seem to be good predictors of stock returns and volatility during the pandemic.

## Introduction

After the World Health Organization (WHO) declared the coronavirus disease (COVID-19) a global pandemic in March 2020, many countries implemented strict quarantine policies that exerted profound effects on economic activity worldwide. Lockdowns have negatively affected all economic sectors, including financial markets, leading to a severe economic crisis (Smales 2021). For example, the total loss in the S&P 500 index, a benchmark stock market index, was 35% in March 2020. Azimli (2020) put the loss in global financial markets in excess of 20% due to COVID-19, and Hashmi et al. (2021) noted that emerging stock markets have been more affected by the global pandemic than developed stock markets.

The effects of the global pandemic on economic activity and financial markets have been examined from various perspectives (Hossain 2021; Sharif et al. 2020; Albulescu 2021; Wei and Han 2021). For instance, Kou et al. (2021) indicated that the use of technology and innovation has increased considerably after the global pandemic to overcome the challenges caused by numerous precautions taken by governments, such as strict quarantine policies. Chundakkadan and Nedumparambil (2021) noted that sharp declines in stock markets are not only due to the lockdowns that restrict economic activities but also due to changes in investor sentiment; as such, there is a growing body of literature that examines the relationship between investor sentiment and stock market behavior during the COVID-19 pandemic. It should be noted that the review of the effects of investor sentiment on the stock market did not start with COVID-19, but such studies gained momentum during the global pandemic. For example, Dergiades (2012) found that changes in investor sentiment help predict returns in the United States. Brown and Cliff (2004) showed that while there is a strong contemporaneous correlation between stock market returns and investor sentiment, such sentiment may contribute little to the prediction of future stock market returns.

The principal objective of the current study is to examine the effects of investor sentiment and mood (positive and negative) on major stock markets during the COVID-19 pandemic. Although a significant body of empirical work examines investor sentiment driven by COVID-19, these studies tend to focus only on negative investor sentiment. Hence, the current study contributes to the existing literature by examining the impact of both negative and positive sentiments due to COVID-19 on stock markets.

Behavioral finance studies show that investors’ emotions and anxiety affect their investment decisions in stock markets; this finding is related to the mood sensitivity hypothesis. However, a problem arises in measuring emotions or investor sentiments because these cannot be observed directly. As such, several proxies have been considered in measuring investor sentiments in the literature. Since the work of Da et al. (2011), the Google Search Volume Index (GSVI) data have been used frequently in the literature to measure investor interest or sentiment. For example, Barber and Odean (2001) noted that the internet has become an essential tool for investors buying and selling decisions in financial markets. Hence, the internet offers investors a vital platform on which they can access comprehensive information for investment decision-making. If an internet search query is considered an indication of direct interest, searching for information on a particular topic on the internet is a clear indication of an individual’s interest in the topic. Da et al. (2011) suggested that investors tend to invest in companies that attract their attention in financial markets. Da et al. (2011) and Fang et al. (2014) examined the effects of internet search volumes on stock returns. Furthermore, Da et al. (2011) indicated that the GSVI data allow us to ascertain investor attention more quickly, as observed during the global pandemic. Similarly, Smales (2021) noted that the GSVI provides a direct and timely measurement of the retrieval of available information. In addition, Costola et al. (2021) emphasized that the GSVI data can successfully gauge investor attention during episodes of diseases, such as the Middle East respiratory syndrome, chickenpox, and flu.

Numerous studies have shown that internet search volumes can be used as a proxy for investor sentiment, highlighting significant relationships between investor sentiment and investment decisions in financial markets (Kamstra et al. 2003; Kaplanski and Levy 2010; Da et al. 2015). Moreover, the effects of investor sentiment on stock market returns have been extensively examined in the literature (Andrei and Hasler 2015; Aouadi et al. 2013; Hirshleifer et al. 2011; Padungsaksawasdi et al. 2019; Chen et al. 2020a; Chemmanur and Yan 2019; Chen 2017; Wen et al. 2019; Han et al. 2018; Smales 2021). In the finance literature, the GSVI is widely used to predict stock returns and volatility (Vlastakis and Markellos 2012; Kim et al. 2019; Heyman et al. 2019).

Chundakkadan and Nedumparambil (2021) showed that “coronavirus” became a trending online query after the COVID-19 outbreak, especially after the WHO declared it a pandemic in March 2020. Volatility in financial markets has increased considerably owing to the longer-than-expected COVID-19 pandemic (Mazur et al. 2021; Zhang et al. 2020; Cheng 2020). Smales (2021) indicated that the continuing uncertainty about the global pandemic is increasing the information needs and news interest of investors, with investor interest playing a key role in the impact of the COVID-19 outbreak on stock markets. To date, there is a significant body of empirical work in which investor sentiment driven by COVID-19 is measured by the GSVI (Chen et al. 2020b; Lyocsa et al. 2020; Chundakkadan and Nedumparambil 2021; Smales 2021; Szczygielski et al. 2021).

Given the panic and fear associated with COVID-19, it is not surprising that internet search queries related to COVID-19 have been used to construct a fear index; hence, empirical studies have mostly examined the effects of negative investor sentiment on stock markets using the GSVI related to COVID-19. Meanwhile, Chundakkadan and Nedumparambil (2021) emphasized that focusing on negative investor sentiment is a limitation of these studies. They noted that some sectors, such as pharmaceuticals and biotechnology have been positively affected by the global pandemic, but it is not easy to distinguish between positive and negative sentiments during this pandemic. For example, Nofsinger (2005) focused on the relationship between investors’ social mood and trading activity and found that an optimistic social mood is related to increases in investment and business activity. Similarly, Shu (2010) found that equity and T-bill prices correlate positively with investor mood, with a good mood leading to an increase in asset prices, which exert a greater effect on equity markets than on the T-bill market. However, this raises the question as to how the good mood of investors related to COVID-19 can be measured given the lack of direct measurement. In the current study, we propose and use internet search queries for COVID-19 vaccines and the names of companies producing these vaccines as a proxy for the good mood of investors because COVID-19 vaccine news in traditional and social media is generally about the development and effectiveness of vaccines; hence, these developments can provide a proxy for positive sentiments related to overcoming the global pandemic. For example, Sattar and Arifuzzaman (2021) and Yousefinaghani et al. (2021) examined tweets on social media and found that the incidence of positive sentiments about COVID-19 vaccines is higher than that of negative sentiments.

**Fig. 1:**
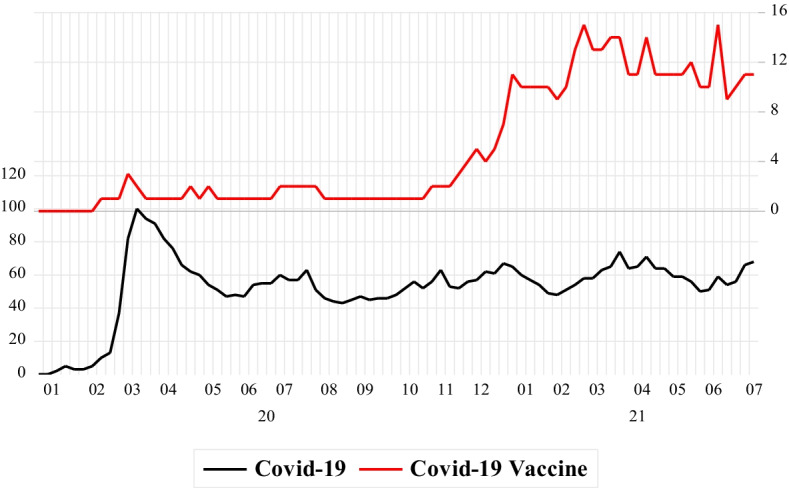
Google Search Volume Index for “COVID-19” and “COVID-19 Vaccine” Terms. *Note* The left axis measures the search volume for “COVID-19” whereas the right axis measures “COVID-19 Vaccine” terms

We present a Google search for the terms “COVID-19” and “COVID-19 Vaccine” in Fig. 1. The results in Fig. 1 clearly show that the internet search for COVID-19 reached its highest value in March 2020, and remained relatively high. This indicates that the anxiety about COVID-19 is still high and that there is a high demand for information about COVID-19. However, the Google search for COVID-19 vaccines was not high until the end of 2020, but it has significantly increased thereafter.

This study contributes to the literature on the effect of investor sentiment (positive and negative) on stock markets in G20 countries by using various estimation methods. We focus on G20 stock markets because G20 countries include major developed and emerging countries and account for approximately 85% of the gross world product and 80% of world trade in goods and services. In addition, the G20 group includes the countries worst affected by COVID-19 in terms of total cases and deaths*.* First, we focus on the Google search for not only COVID-19 but also COVID-19 vaccines to examine negative and positive sentiments related to COVID-19. To the best of our knowledge, this study is the first to explore the effects of positive investor sentiment related to COVID-19 on stock markets. Second, we use the panel quantile estimation method suggested by Machado and Silva (2019) because the relationship between investor sentiment and stock markets may vary over different return and volatility episodes. We also use a panel vector autoregression (PVAR) model to examine the dynamic relationship between positive and negative investor sentiments and stock markets.

To preview our results, we find significant relationships between investor sentiments and stock market returns and volatility. The panel regression model results show that positive and negative investor sentiments affect stock market returns and volatility. Specifically, increases in positive investor sentiments increase stock returns while increases in negative investor sentiments decrease stock returns at lower quantiles according to the panel quantile regression model. The effect of investor sentiment on volatility is consistent across the distribution: negative sentiment increases volatility, whereas positive sentiment reduces volatility. Finally, these results are robust as they are corroborated by the PVAR and time series models.

The rest of the paper is organized as follows. Section 2 provides a brief literature review. Section 3 presents the econometric framework. Section 4 discusses the data and empirical results. Section 5 details the conclusions.

## Literature review

Studies on the impact of the global pandemic have gained significant momentum since the WHO declared COVID-19 a global pandemic in March 2020. The pandemic has adversely affected financial markets by increasing global financial risk (Al-Awadhi et al. 2020; Baker et al. 2020; Cao et al. 2021; Gil-Alana and Claudio-Quiroga 2020; Gormsen and Koijen 2020; Harjoto et al. 2021; Liu et al. 2020b; Phan and Narayan 2020). In addition, empirical studies have found that stock returns have decreased significantly during this period due to the increasing uncertainty caused by the global pandemic (Al-Awadhi et al. 2020; Ambros et al. 2021; Mishra et al. 2020; Topcu and Gulal 2020). Another strand of literature focuses on volatility in financial markets, and the results present clear evidence that the global pandemic has increased volatility in equity markets (Corbet et al. 2020; Haroon and Rizvi 2020a, 2020b; Sharma 2020; Zaremba et al. 2020).

Studies can be classified into three groups. The first group examines the impact of government intervention on economic activity in the wake of the COVID-19 global pandemic. For example, Phan and Narayan (2020) evaluated the effects of government responses to COVID-19 on financial markets. They documented a possible overreaction of stock markets to the pandemic and market corrections over time. Narayan et al. (2021a) examined the impact of government interventions in response to the COVID-19 pandemic, such as lockdowns, stimulus packages, and travel bans, on stock markets in G7 countries. The empirical results show that lockdowns, stimulus packages, and travel bans positively impact stock markets and that the impact of lockdowns is greater than that of the other responses.

Similarly, Bannigidadmath et al. (2021) examined the effects of government policies on stock markets in 25 countries during the global pandemic. They found no significant reaction of stock returns to stimulus packages, lockdowns, or travel bans in Italy, Spain, Belgium, Portugal, Austria, and Sweden. Moreover, they noted that the effects of these policies on stock returns were negative in approximately half of the countries and that stock returns were the least affected by travel bans. Padhan and Prabheesh (2021) surveyed the literature on the impact of the global pandemic on global economic activity. The most effective policies to reduce the adverse effects of the global pandemic are a combination of monetary, macroprudential, and public finance policies. Zaremba et al. (2020) investigated the relationship between government interventions aimed at curbing the spread of COVID-19 and stock market volatility in 67 countries. The results show that non-pharmaceutical interventions significantly increase stock market volatility. Liu et al. (2020a) examined the responses of macro-financial variables in China to COVID-19 by using time–frequency analysis. They found that business and financial cycles were close to recessions before the COVID-19 outbreak. They also indicated that business cycles in China decoupled from global financial cycles after 2015, putting China at an advantage relative to other emerging countries in combating the global pandemic.

The second group of studies in the literature focuses on the relationship between investor sentiment and stock market performance. For example, Wen et al. (2019) examined the impact of retail investor attention, which is measured using the Baidu index as the search frequency, on stock price collapse risk in China. Their empirical results show that an increase in retail investor attention leads to a reduction in future stock price crash risk. Lopez-Cabarcos et al. (2017) investigated the differences between the social media activities of technical and nontechnical investors and their impact on risk in the market. The empirical results indicate that while technical investors’ social media activities have no impact on the perceived risk in the market, the sentiment of nontechnical investors affects market risk. This impact varies according to investors’ profiles, including experience, holding period, and a number of followers. Donadelli et al. (2017) examined the impact of investor sentiment driven by the WHO warnings and media news about dangerous infectious diseases on the stock prices of pharmaceutical companies in the United States. They found that disease-related news positively affected the stock prices of pharmaceutical companies from 2003 to 2014 and that the impact was more substantial on the portfolio of small-capitalization stocks. Ichev and Marinč (2018) examined the relationship between the media coverage of the 2014–2016 Ebola pandemic-related events and the stock prices in the United States in terms of geographical proximity. They found that the impact of the Ebola pandemic was more pronounced on the stock prices of companies operating in West African countries and the United States.

Haroon and Rizvi (2020a) analyzed the relationship between investor sentiment driven by media news related to COVID-19 and the volatility of stock markets. They found that COVID-19-related news causes increased uncertainty in financial markets and increased volatility in stock markets. Ambros et al. (2021) investigated the impact of COVID-19-related news on eight stock market indices. Their empirical results show that while stock returns were not affected by changes in the volume of COVID-19-related news, the volatility of the European stock markets significantly increased due to such news. Iyke and Ho (2021) measured investor attention using Google search terms related to COVID-19 and examined the relationship between investor attention and stock market indices in 14 African countries. They found that investor attention is an important determinant of stock returns, with increases in investor attention decreasing the stock returns in Botswana, Nigeria, and Zambia. Meanwhile, there is a positive relationship between investor attention and stock returns in Ghana and Tanzania. Using word searches from 45 popular newspaper articles, Narayan et al. (2021b) constructed six different global sentiment indicators for COVID-19, namely, COVID, medical, vaccine, travel, uncertainty, and aggregate sentiment. They suggested these indicators provide a good measure for examining the impact of the global pandemic. Piñeiro-Chousa et al. (2022) analyzed the stock market reaction of Pfizer and Moderna, which developed the first vaccines against COVID-19, before and during the pandemic. They considered the impact of the technological market index, market volatility, and investor sentiment on Pfizer and Moderna’s stock returns. They observed that market volatility and investor sentiment exert an asymmetric impact on stock returns. In addition, there is a contagion effect between the stock returns of Pfizer and Moderna and the technological market during the COVID-19 pandemic. Li et al. (2021) suggested a new approach for determining cluster structures for financial data. They showed that the proposed approach performs well in obtaining a reasonable number of cluster structures and in detecting anomalies in financial variables.

Several studies have proxied the effect of the pandemic on stock markets using the total number of cases and deaths due to COVID-19. For example, Al-Awadhi et al. (2020) used panel data analysis to examine the impact of the COVID-19 pandemic on companies traded in the Chinese stock market. They found that the daily increases in total cases and total deaths caused by COVID-19 exerted significant negative effects on the stock returns of all companies considered. Haroon and Rizvi (2020b) investigated the impact of the global pandemic on the liquidity of stock markets in 23 emerging countries. While the decrease in the number of COVID-19 cases positively affects liquidity in financial markets, the increase in cases reduces liquidity. Topcu and Gulal (2020) investigated the impact of COVID-19 on emerging stock markets by using COVID-19 cases and found that although the initial impact of the global pandemic on emerging stock markets was negative, this effect has gradually decreased over time. Cao et al. (2021) analyzed the impact of the COVID-19 pandemic on 14 stock markets by using panel data with the total number of cases as a proxy for the effect of the COVID-19 pandemic. The empirical results show a significantly negative relationship between stock market returns and the total number of cases. Gil-Alana and Claudio-Quiroga (2020) examined the impact of the global pandemic on stock markets in China, Japan, and South Korea. Using fractional integration methods, they found temporary effects of the pandemic on the Japanese stock index but permanent effects on the Chinese and South Korean stock markets. Harjoto et al. (2021) investigated the impact of the global pandemic on stock markets by using an event study approach and found that the global pandemic exerts a greater negative impact on emerging stock markets than on developed stock markets.

The third group of studies in the literature focus on examining COVID-19 vaccine-related sentiments by using data from social media. Sattar and Arifuzzaman (2021) analyzed 1.2 million tweets about COVID-19 vaccines on Twitter to ascertain the effects of the COVID-19 vaccine. In general, they found that the sentiments related to COVID-19 vaccines were positive. Similarly, Yousefinaghani et al. (2021) analyzed approximately 4.5 million tweets to understand the public sentiments and thoughts about COVID-19 vaccines. Their content analysis revealed that positive sentiments about COVID-19 vaccines were dominant. Kwok et al. (2021) analyzed 31,100 tweets, including keywords related to COVID-19 vaccines, by using machine learning methods to determine the effects of COVID-19 vaccine sentiments in Australia. The number of tweets expressing a positive public opinion on COVID-19 vaccines constituted approximately two-thirds of the total tweets. Hussain et al. (2021) analyzed posts about COVID-19 vaccines on social media by using an artificial intelligence approach to ascertain public attitude and concerns regarding COVID-19 vaccines in the United Kingdom and the United States. They concluded that the overall mood of vaccine-related tweets and Facebook posts in the two countries were positive.

The empirical results in the literature show that investor sentiment affects the stock market. Therefore, we examine the relationship between investor sentiment (positive and negative) and the stock market on the basis of the following hypotheses:

### H1

There is a significant and negative (positive) relationship between negative (positive) investor sentiment and stock returns.

### H2

There is a significant and positive (negative) relationship between negative (positive) investor sentiment and stock market volatility.

### H3

The effects of investor sentiment on returns and volatility are heterogeneous across the distribution of returns and volatility.

## Econometric framework

### Model specification

Behavioral finance studies have shown that investors’ emotions and anxiety affect their investment decisions in stock markets; this finding is related to the mood sensitivity hypothesis. As such, the literature has extensively focused on the relationship between investor sentiment and stock returns and volatility (e.g., Dergiades 2012; Brown and Cliff 2004; Da et al. 2011; Fang et al. 2014; Smales 2021). In this context, Chundakkadan and Nedumparambil (2021) noted that sharp declines in stock markets during the COVID-19 pandemic are not only due to lockdowns that restrict economic activity but also due to changes in investor sentiment. On the basis of behavioral finance, we examine the effects of investor sentiment on stock returns and volatility by using the following panel regression models:

1 $$CAR_{it} = \alpha_{i0} + \beta_{1} COV19_{it} + \beta_{2} VAC_{it} + \beta_{3} X_{it} + \varepsilon_{it}$$

2 $$VOL_{it} = \alpha_{i0} + \beta_{1} COV19_{it} + \beta_{2} VAC_{it} + \beta_{3} X_{it} + \varepsilon_{it}$$

where *CAR* and *VOL* are the cumulative abnormal returns and realized volatility, respectively; *COV19 and VAC* are the GSVIs for the COVID-19- and COVID-19 vaccine-related terms, respectively; and *X* is the vector of the control variables.^1^ To estimate Eqs. (1) and (2), we use a fixed effect regression model with Driscoll and Kraay standard errors that produce robust standard errors in the case of cross-sectional dependence (CD) and autocorrelation.

### Panel quantile regression model

Note that the results of the fixed effect panel regression model provide only the mean effects of investor sentiment on returns and volatility; however, these effects may be heterogeneous across the entire distribution of returns and volatility. The quantile regression model suggested by Koenker and Bassett (1978) is preferred in examining the heterogeneous effects of investor sentiments. Since the report of Koenker and Bassett (1978), the quantile regression model has been widely used in the empirical literature because it allows for examining the effect of the exogenous variables on the conditional mean of the dependent variable at different quantiles. In addition, quantile regressions provide more robust estimation results in the case of outliers and non-normal data. In this study, we use panel quantile estimation methods with fixed effects; namely, the method of moments quantile regression (MMQR) suggested by Machado and Silva (2019).

Controlling for unobserved individual heterogeneity is the most important issue in estimating the quantile model for panel data; hence, the fixed effects panel quantile regression is widely used in considering unobserved individual heterogeneity. Machado and Silva (2019) emphasized that the most important advantage of the MMQR approach is that it provides additional information on how explanatory variables affect the entire conditional distribution of the dependent variable. This is in contrast to other methods in the literature, such as that by Koenker (2004) and Canay (2011), in which the estimated coefficients of independent variables provide an idea about the conditional mean response of the dependent variable. Therefore, the MMQR approach allows for examining the effects of individual heterogeneity on the entire distribution. Additionally, the MMQR approach can be used when there are endogenous variables on the right-hand side.

Given the data $\left\{ {\left( {Y_{it} ,X_{it}^{\prime } } \right)^{\prime } } \right\}$ from a panel of n entities *i* = 1, …, *n* over *T* periods, *t* = 1, …, *T*, the estimation of the conditional quantiles $Q_{y} \left( {\tau \backslash X} \right)$ for a location-scale model of the form can be presented as follows:

3 $$Y_{it} = \alpha_{it} + X_{it}^{^{\prime}} \beta + \left( {\delta_{it} + Z_{it}^{^{\prime}} \gamma } \right)U_{it}$$

where $Pr\left\{ {\delta_{it} + Z_{it}^{^{\prime}} \gamma > 0} \right\} = 1$ and $\left( {\alpha \beta^{\prime}\delta \gamma^{\prime}} \right)$ are the estimated parameters. Individual fixed effects are represented by $\left( {\alpha_{i} ,\delta_{i} } \right)$, *i* = 1, …, *n*. *X*_*it*_ is strictly exogenous *i.i.d.* for any fixed *i* and is independent across *i*. *U*_*it*_ is *i.i.d.* across individuals (*i*) through time (*t*), orthogonal to *X*_*it*_, and normalized to satisfy the moment conditions given in the work of Machado and Silva (2019). *Z* is a vector of the identified components of *X*, which are differentiable transformations with element *l* given by

4 $$Z_{l} = Z_{l} \left( X \right), l = 1, \ldots ,k$$

Equation (4) can be represented as follows:

5 $$Q_{y} \left( {\tau \backslash X_{it} } \right) = \left( {\alpha_{i} + \delta_{i} q\left( \tau \right)} \right) + X_{it}^{^{\prime}} \beta + Z_{it}^{^{\prime}} \gamma q\left( \tau \right)$$

where $X_{it}^{^{\prime}}$ is a vector of independent variables and $Q_{y} \left( {\tau \backslash X_{it} } \right)$ is the quantile distribution of the dependent variable *Y*_*it*_, which is conditional on the location of the independent variables. Note that $\alpha_{i} \left( \tau \right) \equiv \alpha_{i} + \delta_{i} q\left( \tau \right)$ is the scalar coefficient, which is indicative of the quantile-τ fixed effect for the individual *I* and $q\left( \tau \right)$ is the *τ*-*th* sample quantile.

## Panel VAR Model

The fixed effects panel regression model and fixed effects panel quantile regression allow us to examine the static relationship between investor sentiment and stock markets; however, the relationship may be dynamic. To account for this possibility, we use a PVAR model to examine the dynamic relationship between investor sentiment and stock markets. Abrigo and Love (2016) put forth the following homogenous PVAR of order *p* with panel-specific effects for *k* variables:

6 $$\begin{array}{*{20}c} {Y_{it} = Y_{it - 1} A_{1} + Y_{it - 2} A_{2} + \ldots + Y_{it - p} A_{p} + X_{it} B + u_{i} + e_{it} } \\ {i \in \left\{ {1,2, \ldots ,N} \right\}, t \in \left\{ {1,2, \ldots ,T} \right\}} \\ \end{array}$$

where *Y*_*it*_ is a vector of endogenous variables, *X*_*it*_ is a vector of exogenous covariates, and *u*_*i*_ and *e*_*it*_ are vectors of dependent variable-specific panel fixed effects and idiosyncratic errors, respectively. *A* and *B* are parameter matrices. The properties of residuals can be described as $E\left( {e_{it} } \right) = 0$, $\sum = E\left( {e_{it}^{^{\prime}} e_{it} } \right)$ and $E\left( {e_{it}^{^{\prime}} e_{it} } \right) = 0$ for all *t* > *s*.

Abrigo and Love (2016) suggested using fixed effects in estimation to account for cross-sectional heterogeneity. Note that Eq. (6) cannot be estimated using ordinary least squares because the presence of lagged dependent variables on the right-hand side of the system of equations may yield biased results when *N* is large. Abrigo and Love (2016) suggested that generalized method of moments (GMM) estimations provide consistent estimates for the PVAR model when *T* is fixed and *N* is large. The most important issue in GMM estimation is avoiding the over-identification problem. Abrigo and Love (2016) indicated that the *J* test suggested by Hansen (1982) can be used to ascertain over-identifying restrictions for instrumental variables.

As in the time series VAR model, selecting the optimal lag length is the most important task in the PVAR model. There are three popular model selection criteria: the Akaike information criterion (AIC), Bayesian information criterion (BIC), and Hannan–Quinn information criterion (HQIC). Similarly, Abrigo and Love (2016) suggested a panel version of the three model selection criteria, namely, modified AIC (MAIC), modified BIC, and modified HQIC; this version depends on the *J*-statistic to determine optimal lag lengths.

## Data and empirical results

### Data

In this study, we examine the effects of investor sentiment on G20 stock markets (minus the aggregate European Union) by using weekly data from March 13, 2020, to May 21, 2021.^2^ We consider five-day cumulative abnormal returns and realized volatility as indicators of stock market performance. We calculate the realized variance for each stock market index using the sum of squared daily returns. We consider the changes in COVID-19 cases to gauge the impact of the global pandemic. We also follow the work of Hood and Malik (2003), Humpe and McMillan (2009), Jain and Biswal (2016), and Ma et al. (2021) and use the 10-year government bond yield, logarithmic changes in the foreign exchange rate,^3^ and logarithmic changes in the gold price^4^ as control variables that affect stock market returns and volatility. The data for the stock market index, gold price, and foreign exchange rate are obtained from Refinitiv Eikon Datastream. The data for investor sentiment are derived from Google Trends, and the COVID-19 cases data are collected from the Our World in Data database.^5^

In this study, we use cumulative abnormal returns rather than returns because Gao et al. (2017) emphasized that using the former removes market-wide effects. As in Liu et al. (2020b), we first calculate expected returns by using the following market model:

7 $$R_{i,t} = \alpha + \beta R_{m,t} + \varepsilon_{it}$$

where *R*_*it*_ is the daily log return for each country and *R*_*mt*_ is the daily log return for the market. In this study, we consider the MSCI World Index as a benchmark and employ recursive rolling estimation with a rolling window of 252 to obtain the following time-varying abnormal returns:

8 $$AR_{i,t} = R_{i,t} - \hat{\alpha } - \hat{\beta }R_{m,t}$$

where *AR* is the daily abnormal return. Finally, we calculate the weekly cumulative abnormal returns (*CAR*) as the sum of the five-day abnormal returns over one week.

As in Lyocsa et al. (2020), Chen et al. (2020b), Szczygielski et al. (2021), and Smales (2021), we consider country-specific Google search terms “COVID-19,” “Coronavirus,” “Pandemic,” “SARS-CoV,” and “SARSCoV-2” to proxy for negative investor attention to the global pandemic. In addition to the COVID-19 vaccine-related terms, we include the names of the companies producing COVID-19 vaccines to construct country-specific positive investor sentiment. Hence, “COVID-19 Vaccine,” “BioNTech,” “Pfizer,” “Moderna,” “AstraZeneca,” “Johnson & Johnson,”, “Sputnik V,” “Sinovac Biotech,” “Novavax,” and “CanSino Biologics” are used as the Google search terms to proxy for positive investor attention related to COVID-19. The weekly aggregate index is calculated as the sum of the search terms for each week.

Note that we use the Google search volume for the COVID-19 terms to proxy for negative investor sentiment and the Google search volume for COVID-19 vaccine terms to proxy for positive investor sentiment.

We present the definition of the variables in Table 1.

**Table 1:** Description of variables

Variables	Definition
CAR	Cumulative abnormal returns
LVOL	Logarithm of realized volatility
COV-19	Google search volume index for the Covid-19 terms
VAC	Google search volume index for the Covid-19 vaccine terms
CASES	Logarithmic change in the total Covid-19 cases
FX	Logarithmic change in the foreign exchange rate
GOLD	Logarithmic change in the gold price
BOND	10-year government bond yield

### Empirical results

Descriptive statistics are presented in Table 2. According to the results in Table 2, the weekly mean of cumulative abnormal returns is negative during the COVID-19 pandemic. The highest abnormal return occurs in the Argentinean stock market, whereas the lowest one occurs in the Brazilian stock market during the sample. Note that the mean of positive investor sentiment is higher than that of negative investor sentiment and that Brazil has the highest Google search volume for COVID-19 terms. The mean of the changes in the total number of COVID-19 cases is positive during the sample period. In addition, the mean foreign exchange returns and mean gold returns are positive, indicating that the foreign exchange rate and gold provide positive yields during the pandemic.

**Table 2:** Descriptive statistics

	Mean	Std. Dev	Min	Max
CAR	− 0.893	6.008	− 35.352	24.651
LVOL	2.211	1.299	− 4.590	6.881
COV-19	26.676	22.711	4	173
VAC	79.948	74.772	0	358
CASES	0.099	0.472	− 2.037	4.259
FX	0.008	1.717	− 10.443	11.115
GOLD	0.182	2.559	− 14.089	10.575
BOND	3.779	3.762	− 0.638	18.16

The Pearson correlation coefficients are presented in Table 3. Realized volatility, negative investor sentiment, gold returns, and bond yields are negatively and significantly correlated with stock returns. On the contrary, the correlation between stock returns and positive investor sentiment is positive and statistically significant. While the correlation between realized volatility and negative investor sentiment is positive and significant, the relationship between realized volatility and positive investor sentiment is negative. These findings suggest that the Google search for COVID-19-related terms leads to negative investor sentiment because there is a negative (positive) relationship between stock market returns (volatility) and the GSVI. This result is consistent with the literature because as noted above, the Google search volume for COVID-19 has been used to construct a “fear index.” Meanwhile, the positive (negative) and statistically significant relationship between the GSVI for COVID-19 vaccine-related terms and stock market return (volatility) indicates that the Google search for COVID-19 vaccine-related terms can be used as a proxy for positive investor sentiment.

**Table 3:** Correlations among the variables

	CAR	LVOL	COV-19	VAC	CASES	FX	GOLD	BOND
CAR	1.000							
LVOL	− 0.118***	1.000						
COV-19	− 0.127***	0.454***	1.000					
VAC	0.074***	− 0.195***	− 0.220***	1.000				
CASES	− 0.089***	0.388***	0.528***	− 0.140***	1.000			
FX	0.086***	0.181***	0.199***	− 0.022	0.196***	1.000		
GOLD	− 0.189***	0.023	0.097***	− 0.001	0.046	0.341***	1.000	
BOND	− 0.094***	0.221***	− 0.093***	0.035	0.096***	0.093***	0.061**	1.000

^***^, **, and * indicate statistically significant correlations at the 1%, 5%, and 10% levels, respectively

We begin our empirical analysis by first investigating the presence of CD within the panel. To this end, we use the CD test suggested by Pesaran (2015). The CD test results are essential for selecting appropriate panel unit root tests. First-generation unit root tests are known to have low power in rejecting the null hypothesis when CD exists across panel members. Hence, we use both the first- and second-generation panel unit root tests such as Levin-Lin-Chu (LLC) and cross-sectional augmented Im-Pesaran-Shin (CIPS) suggested by Levin et al. (2002) and Pesaran (2007), respectively. According to the test results in Table 4, the null hypothesis of weak CD is rejected at the 1% significance level for all variables, implying strong CD across the countries in the panel. Moreover, the panel unit root test results in Table 4 show that the null hypothesis of a unit root can be rejected at the 1% significance level, indicating that all the variables are stationary.

**Table 4:** Descriptive statistics

	CD test	LLC	CIPS
CAR	63.343 [0.000]	− 50.662***	− 6.190***
LVOL	90.453 [0.000]	− 23.631***	− 6.111***
COV-19	95.119 [0.000]	− 20.351***	− 3.457***
VAC	88.836 [0.000]	− 6.400***	− 3.942***
CASES	41.924 [0.000]	− 14.782***	− 4.673***
FX	45.474 [0.000]	− 39.250***	− 5.733***
GOLD	78.545 [0.000]	− 35.455***	− 6.128***
BOND	62.666 [0.000]	− 1.790**	− 6.355***

The numbers in square brackets are *p*-values. *** indicates stationarity at the 1% significance level

After confirming stationarity, we use a fixed-effects panel regression model with Driscoll–Kraay standard errors and present the model results in Table 5.^6^ According to the results in Table 5, an increase in negative investor sentiment significantly decreases stock returns; this relationship is consistent with those described in established studies (Chen et al. 2020b; Chundakkadan and Nedumparambil, 2021; Smales, 2021; Szczygielski et al., 2021). The estimated coefficient for positive investor sentiment is positive and statistically significant at the 10% level. Hence, it can be said that the Google search index for COVID-19 vaccine-related terms significantly affects stock market returns during the COVID-19 pandemic.

**Table 5:** Fixed effects panel regression results

	Dependent variable: CAR			Dependent variable: LVOL		
	Coefficient	Std. Errors	*p*-value	Coefficient	Std. Errors	*p*-value
Constant	0.873	1.620	0.592	1.123	0.221	0.000
COV-19	− 0.030	0.014	0.041	0.025	0.003	0.000
VAC	0.006	0.003	0.091	− 0.003	0.001	0.004
CASES	− 0.571	0.554	0.307	0.302	0.114	0.010
FX	0.741	0.407	0.074	0.036	0.042	0.389
GOLD	− 0.566	0.286	0.052	− 0.035	0.025	0.172
BOND	− 0.342	0.396	0.391	0.172	0.066	0.012
R^2^	0.090			0.371		
F-stat	2.66 [0.023]			25.94 [0.000]		
Time Effect	No			No		

Interestingly, we find no statistically significant relationship between COVID-19 cases and stock market returns. However, an increase in gold returns and bond yields decreases stock market returns. In addition, foreign exchange returns positively affect stock market returns.

Looking at the results for stock market volatility, an increase in negative investor sentiment increases volatility in stock markets; this relationship is consistent with the results in Lyocsa et al. (2020), Chundakkadan and Nedumparambil (2021), and Smales (2021). On the other hand, positive investor sentiment significantly reduces stock market volatility. Moreover, stock market volatility reacts positively to the number of COVID-19 cases, where an increase in COVID-19 cases leads to an increase in volatility. It should be noted that the impact of investor sentiment on stock market volatility is stronger than the effect on stock market returns because the coefficients for positive and negative investor sentiments are statistically significant at the 1% level in the stock market volatility model. Therefore, stock market volatility seems to be more sensitive to the volume of Google search terms related to COVID-19.

The panel regression model results show that positive and negative investor sentiments affect stock market returns and volatility. Meanwhile, the mean effects of investor sentiment may be heterogeneous across the distribution of returns and volatility. To account for this possibility, we use a fixed-effects panel quantile regression model and present the results in Table 6, where the results for stock market returns and volatility are shown in Panels A and B, respectively. While the results in the “Location” column in Table 6 give the mean effect of independent variables on the dependent variable, the results in the “Scale” column show the effect of independent variables on the dispersion of the dependent variable. The estimated coefficients for positive and negative investor sentiments are statistically significant in both models per the “Location” and are consistent with the results in Table 5. According to the results in Panel A, the measure for negative investor sentiment has a positive impact on the scale, implying that an increase in negative sentiment leads to an increase in the dispersion of stock returns. On the contrary, the negative coefficient for positive sentiment indicates that an increase in positive investor sentiment is accompanied by a decrease in the dispersion of stock returns. In addition, while positive investor sentiment does not seem to exert a significant impact on volatility dispersion, negative investor sentiment increases volatility dispersion.

**Table 6:** Fixed-effects panel quantile regression results

Dependent variable: CAR			Quantiles								
	Location	Scale	0.1	0.2	0.3	0.4	0.5	0.6	0.7	0.8	0.9
*Panel A*											
Constant	0.873	2.407**	− 2.982	− 1.610	− 0.740	0.060	0.849	1.648	2.305*	3.215**	4.656***
COV-19	− 0.030**	0.038***	− 0.091***	− 0.069***	− 0.056***	− 0.043***	− 0.030**	− 0.018	− 0.007	0.006	0.029*
VAC	0.006***	− 0.010***	0.022***	0.016***	0.013***	0.009***	0.006***	0.002	0.001	− 0.003	− 0.010***
CASES	− 0.571	0.445	− 1.284*	− 1.030*	− 0.869	− 0.721	− 0.575	− 0.427	− 0.362	− 0.137	0.128
FX	0.741***	0.013	0.720***	0.728***	0.732***	0.737***	0.741***	0.476***	0.749	0.754***	0.762***
GOLD	− 0.566***	0.045	− 0.638***	− 0.613***	− 0.596***	− 0.581***	− 0.567***	− 0.551***	− 0.539	− 0.522***	− 0.495***
BOND	− 0.342	0.381	0.953	− 0.735	− 0.598	− 0.471	− 0.346	− 0.219	− 0.115	0.028	0.256

Dependent variable: LVOL			Quantiles								
	Location	Scale	0.1	0.2	0.3	0.4	0.5	0.6	0.7	0.8	0.9
*Panel B*											
Constant	1.123***	0.657***	0.065	0.426**	0.710***	0.984***	1.162***	1.350***	1.550***	1.831***	2.164***
COV-19	0.024***	0.002**	0.020***	0.022***	0.023***	0.023***	0.024***	0.025***	0.026***	0.027***	0.028***
VAC	− 0.003***	0.001	− 0.003***	− 0.003***	− 0.003***	− 0.003***	− 0.003***	− 0.002***	− 0.002***	− 0.002***	− 0.002***
CASES	0.302***	− 0.029	0.350***	0.333***	0.321***	0.310***	0.301***	0.292***	0.283***	0.271***	0.256**
FX	0.036*	− 0.012	0.056**	0.049**	0.044**	0.040*	0.036*	0.032*	0.028	0.023	0.017
GOLD	1.123***	0.657***	0.065	0.426**	0.710***	0.984***	1.162***	1.350***	1.550***	1.831***	2.164***
BOND	0.024***	0.002**	0.020***	0.022***	0.023***	0.023***	0.024***	0.025***	0.026***	0.027***	0.028***

Standard errors are calculated using Monte Carlo simulation with 1000 repetitions. ***, **, and * indicate statistically significant coefficients at the 1%, 5%, and 10% levels, respectively

The results in Panel A show that while the impact of negative and positive investor sentiments on stock market returns is statistically significant between the 1st and 5th quantiles, it is not significant at the higher quantiles, except for the 9th quantile. Thus, an increase in positive (negative) investor sentiment leads to an increase (decrease) in stock returns up to the median of stock returns. The lack of a significant relationship at higher quantiles implies that the effect of investor sentiment on stock returns is significant only on the left-hand side of the distribution. This implies that investor sentiment has a strong impact on stock returns under bad market conditions. This result is consistent with Ma et al. (2018), who used a different proxy for investor sentiment and showed that investor sentiment contains significant information about the left tail of market returns.

Similarly, Li et al. (2017) documented Granger causality from investor sentiment to stock returns at low quantiles. Note that positive investor sentiment has a significant negative impact on stock returns at the highest quantile, which is consistent with the prospect theory suggested by Kahneman and Tversky (1979). Kahneman and Tversky (1979) indicated that losses are more important to people than gains. In this vein, Li et al. (2017) emphasized that investors tend to be prudent or hesitant in making investment decisions in expansionary market regimes because losses may be large if market conditions change. Therefore, investors may take short positions at the highest return levels even if they have positive sentiments. Hence, positive investor sentiment negatively affects stock returns.

The effect of the total number of COVID-19 cases on stock returns is statistically significant only at 1st and 2nd quantiles, suggesting that the impact of the total number of COVID-19 cases on stock returns is limited. While the effects of foreign exchange rates and gold returns on stock returns are statistically significant across all quantiles, the estimated coefficient for bond yields is not significant.

**Fig. 2:**
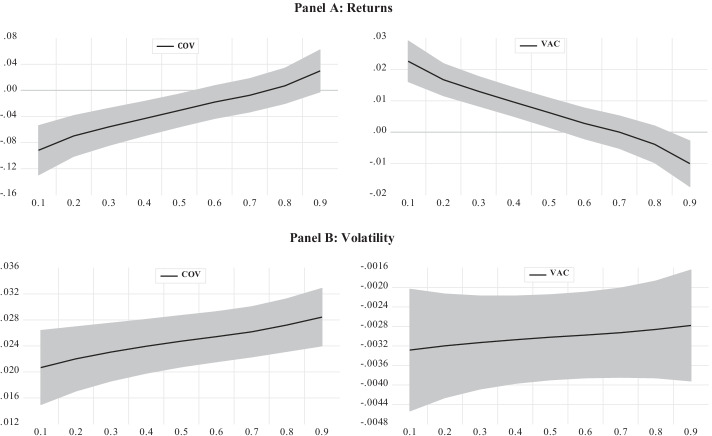
Impact of positive and negative investor sentiments on returns and volatility. *Note* The shaded areas are two standard deviation confidence intervals

The model results in Panel B show that the effects of positive and negative investor sentiments on stock market volatility are consistent across all quantiles. Specifically, the estimated coefficients for negative investor sentiment are negative and statistically significant across all quantiles, where negative investor sentiment increases volatility. Note that the effect of negative investor sentiment on volatility increases slightly in higher quantiles. We find robust evidence that positive investor sentiment reduces volatility because the estimated coefficients for positive investor sentiment are negative and statistically significant across the quantiles. Moreover, our empirical findings show that increases in the total number of COVID-19 cases contribute to stock market volatility. We also find that foreign exchange rates, gold returns, and bond yields positively affect stock market volatility as their coefficients are all positive and statistically significant. These results highlight the importance of government policy responses. For example, Goel and Dash (2022) found that government policy responses, as measured by various pandemic response policies, play a moderating role in the relationship between investor sentiment and stock returns. It is also important for government health policies to rapidly communicate accurate information about COVID-19 to mitigate the effects of the pandemic on financial markets. To better illustrate the effects of positive and negative investor sentiments on stock market returns and volatility across quantiles, we present the estimated coefficients for positive and negative investor sentiments in Fig. 2.

Figure 2 clearly shows that the effect of investor sentiment on returns is statistically significant up to the median of returns with limited effects at higher return levels. The results in Panel B indicate that the impact of investor sentiment on stock market volatility is statistically significant across all quantiles. More interestingly, the negative and positive effects of investor sentiment on stock market volatility are stronger at higher volatility levels.

### Robustness analysis

In this section, we employ two robustness checks. First, we use a PVAR model to ascertain the dynamic relationship between stock markets (returns and volatility) and investor sentiment. Second, we estimate a country-specific regression model to examine whether the relationship between stock market returns and investor sentiment varies by country.

### PVAR model results

We first set the optimal lag length for the PVAR model.^7^ We consider model information criteria and Hansen’s *J* test for overidentification; the MAIC model and Hansen’s highest *J* test suggest that one lag is sufficient. Therefore, we consider one lag in the PVAR estimation.^8^

The dynamic relationships among the variables can be analyzed using Granger causality and impulse response analysis in the PVAR model. Therefore, we use the Wald test by imposing zero restrictions on the estimated autoregressive coefficients to analyze investor sentiment, stock market returns, and volatility. Table 7 presents the Granger causality test results. The results in Table 7 show unidirectional Granger causality from negative investor sentiment to stock returns. The null hypothesis of no causality between positive investor sentiment and stock returns can be rejected at the 5% significance level.

**Table 7:** Panel granger causality test results

Null Hypothesis	Test stat	*p*-value
COV-19 does not cause CAR	12.150	0.000
CAR does not cause COV-19	0.410	0.522
VAC does not cause CAR	4.980	0.026
CAR does not cause VAC	0.809	0.368
COV-19 does not cause LVOL	54.171	0.000
LVOL does not cause COV-19	1.791	0.181
VAC does not cause LVOL	25.006	0.000
LVOL does not cause VAC	10.950	0.001

^***^, **, and * indicate the presence of causality relation at the 1%, 5%, and 10% significance levels, respectively

However, we cannot find causality from stock returns to negative investor sentiment. While unidirectional Granger causality exists only from negative investor sentiment to stock market volatility, bidirectional causality exists between positive investor sentiment and stock market volatility. Overall, the Granger causality test results show that investor sentiment plays an important role in predicting stock market returns and volatility during the COVID-19 pandemic which has important portfolio allocation implications.

**Fig. 3:**
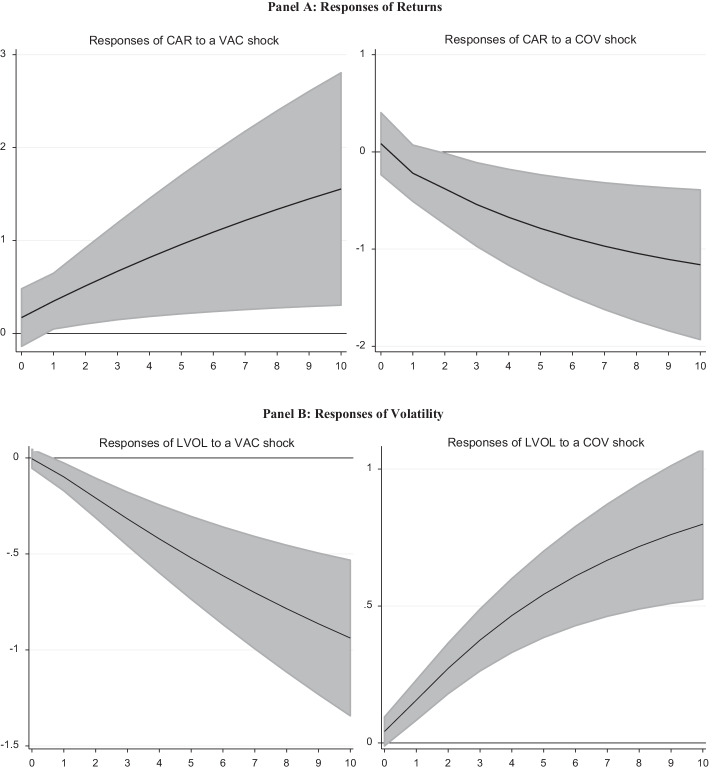
Impulse Response Analysis Results. *Note* The figures show the cumulative orthogonalized impulse response functions. The shaded areas represent two standard deviation confidence intervals. Moreover, the shaded areas represent 95% confidence intervals. A Monte Carlo simulation with 1,000 draws is used to obtain the confidence intervals

The impulse response analysis results are shown in Fig. 3.^9^ Note that the results in Fig. 3 are the cumulative responses of returns and volatility to a one standard deviation shock in investor sentiment. According to the results in Panel A, stock returns react positively to a shock in positive investor sentiment and are statistically significant for up to 10 weeks. The responses of stock returns to negative investor sentiment shocks are negative and statistically significant. This finding is consistent with Granger causality where we find a causal link between investor sentiment and stock returns. These findings also have portfolio implications as measures of investor sentiment seem to have predictive power for stock returns and volatility during the pandemic.

The responses of stock market volatility to positive investor sentiment are negative and significant. Meanwhile, stock market volatility increases due to negative investor sentiment during the COVID-19 pandemic. The PVAR model results are consistent with those of the panel regression and quantile models presented above and tell a consistent story: the Google search volume for COVID-19-related terms negatively affects stock markets in the sample. At the same time, the Google search terms for COVID-19 vaccine-related terms and the prospect of an end to the pandemic positively affect stock markets in the G20 countries. Thus, Google search terms seem to be good proxies for investor sentiment.

### Country-specific results

A country-specific regression analysis allows us to examine how each stock market responds to investor sentiment. The time-series results for stock market returns are presented in Table 8.^10^ The results in Table 8 show that while the impact of negative investor sentiment on stock market returns is negative for all countries, except for China, India, Japan, and Saudi Arabia, it is statistically significant for Australia, Canada, France, Germany, Italy, Mexico, Russia, the United Kingdom, and the United States. Meanwhile, the regression model results for positive investor sentiment are mixed. For example, stock returns seem to be negatively affected by positive investor sentiment in Australia, China, Indonesia, Japan, Mexico, Russia, Turkey, and the United Kingdom. However, the coefficient of positive investor sentiment is statistically significant at the 10% level only for Japan and Saudi Arabia. These results may be due to the low number of observations (i.e., 66 observations) for each country; hence, the estimated coefficients may be insignificant because of low degrees of freedom.

**Table 8:** Country Regression Model Results for Cumulative Abnormal Returns (CAR)

Dependent Variable: CAR						
Countries	Constant	COV-19	VAC	CASES	R^2^	F-stat
Argentina	75.538*	− 0.091	0.014	3.159	0.084	0.865
Australia	3.622**	− 0.098***	− 0.004	− 0.487	0.372	5.548***
Brazil	− 6.616	− 0.062	0.010	− 1.508	0.178	2.030*
Canada	2.871	− 0.144***	0.016	2.995	0.439	7.322***
China	− 7.245	0.007	− 0.007	0.536	0.080	0.815
France	1.688	− 0.096**	0.003	− 0.208	0.241	2.973**
Germany	5.198	− 0.150**	0.004	4.035*	0.258	3.247***
India	− 14.396	0.043	0.007	− 7.949***	0.272	3.487***
Indonesia	− 1.235	− 0.029	− 0.002	− 1.029	0.276	3.560***
Italy	2.620	− 0.215***	0.003	3.614	0.277	3.578***
Japan	− 0.274	0.018	− 0.010*	− 2.968*	0.311	4.231***
Mexico	− 6.035	− 0.113***	− 0.006	− 1.921	0.330	4.615***
Russia	9.880	− 0.347**	− 0.005	14.188	0.197	2.291**
S. Africa	− 10.091	− 0.074	0.001	− 0.789	0.112	1.187
S. Arabia	− 0.941	0.027	0.006*	− 2.755	0.187	2.623**
S. Korea	2.406	− 0.018	0.003	− 0.272	0.152	1.684
Turkey	− 7.898	− 0.045	− 0.016	− 0.511	0.514	9.887***
UK	0.897	− 0.093**	− 0.001	0.450	0.256	3.214***
US	4.295**	− 0.213***	0.002	3.456*	0.614	17.481***

The standard errors are calculated using the Newey–West variance–covariance matrix. ***, **, and * indicate statistically significant coefficients at the 1%, 5%, and 10% levels, respectively

The results in Table 9 show that stock market volatility is positively and significantly affected by negative investor sentiment in all countries, except Brazil and Turkey. On the contrary, positive investor sentiment leads to decreased volatility in all countries, except Argentina, Japan, Russia, and South Korea. However, the estimated coefficient for positive investor sentiment is statistically significant only in Australia, France, Germany, India, Italy, Mexico, and the United Kingdom.

**Table 9:** Country regression model results for volatility (LVOL)

Dependent variable: LVOL						
Countries	Constant	COV-19	VAC	CASES	R^2^	F-stat
Argentina	10.516**	0.023***	0.001	0.501*	0.314	4.288***
Australia	1.149**	0.032***	− 0.004*	− 0.101	0.512	9.739***
Brazil	4.789***	0.007	− 0.001	1.718***	0.620	15.255***
Canada	0.362	0.043***	− 0.005	− 0.729	0.605	14.342***
China	− 0.689	0.024***	− 0.001	− 0.225**	0.229	2.787**
France	2.110***	0.031***	− 0.009***	− 0.073	0.570	12.392***
Germany	2.678***	0.035**	− 0.008***	− 0.145	0.377	5.654***
India	− 3.391	0.022***	− 0.003**	1.235*	0.608	14.483***
Indonesia	1.319	0.027***	− 0.001	0.498	0.480	8.633***
Italy	0.485	0.027**	− 0.004*	0.417	0.591	13.495***
Japan	1.064***	0.012***	0.001	0.545	0.203	2.382**
Mexico	0.470	0.014**	− 0.003**	0.371	0.535	10.760***
Russia	1.778	0.039***	0.001	0.014	0.536	10.798***
S. Africa	0.031	0.018**	− 0.002	0.305**	0.570	12.387***
S. Arabia	0.347	0.010**	0.001	1.871***	0.491	11.034***
S. Korea	3.047***	0.015***	0.003	− 0.584**	0.308	4.164***
Turkey	0.965	0.006	− 0.001	0.550***	0.316	4.317***
UK	1.598***	0.034***	− 0.005***	0.013	0.477	8.515***
US	1.021	0.029*	− 0.004	0.636	0.477	10.455***

The standard errors are calculated using the Newey–West variance–covariance matrix. ***, **, and * indicate statistically significant coefficients at 1%, 5%, and 10% levels, respectively

Country-specific regression results show that the mean effect of investor sentiment may be heterogeneous across the distributions of returns and volatility. To account for this possibility, we estimate the quantile regression models for each country and present the results for the stock market returns in Table 10. The estimated coefficients for negative investor sentiment are negative and statistically significant at certain quantiles in Australia, Canada, France, Germany, India, Indonesia, Italy, Mexico, South Korea, the United Kingdom, and the United States. At the same time, the results in Table 10 indicate a positive and significant relationship between stock market returns and positive investor sentiment at the lowest quantiles in Argentina, Canada, France, Germany, Italy, and Saudi Arabia.

**Table 10:** Country quantile regression results for stock returns

Dependent variable: CAR										
	Argentina		Australia		Brazil		Canada		China	
	COV-19	VAC	COV-19	VAC	COV-19	VAC	COV-19	VAC	COV-19	VAC
0.1	− 0.408	0.065***	− 0.135***	− 0.003	− 0.092	0.029	− 0.244***	0.054*	0.038	− 0.002
0.2	− 0.423	0.048*	− 0.138***	− 0.001	− 0.056	0.044	− 0.222***	0.047*	− 0.068	− 0.003
0.3	− 0.031	0.04*	− 0.101	0.002	0.017	0.03	− 0.183*	0.019	0.056	− 0.008
0.4	− 0.047	0.025	− 0.046	0.001	0.086	0.021	− 0.187*	0.023	0.078	− 0.01
0.5	0.06	0.015	− 0.085***	− 0.007	0.015	0.002	− 0.165**	0.012	0.047	− 0.011
0.6	0.184	0.001	− 0.091***	0.003	− 0.035	− 0.01	− 0.137	0.005	0.08	− 0.008
0.7	0.133	− 0.004	− 0.096***	− 0.006	0.052	− 0.013	− 0.07	− 0.008	0.026	− 0.001
0.8	0.069	− 0.011	− 0.086*	− 0.023*	− 0.097	− 0.007	− 0.086	− 0.011	0.045	− 0.003
0.9	− 0.112	− 0.059*	− 0.016	0.02	− 0.376	0.027	0.022	0.017	0.083	− 0.009

	France		Germany		India		Indonesia		Italy	
	COV-19	VAC	COV-19	VAC	COV-19	VAC	COV-19	VAC	COV-19	VAC
0.1	− 0.252	0.028***	− 0.196***	0.027**	− 0.029	0.013	− 0.07	− 0.004	− 0.364***	0.006
0.2	− 0.125*	0.018	− 0.209***	0.025*	− 0.014	0.017	− 0.026	− 0.004	− 0.264***	0.021*
0.3	− 0.122	0.018	− 0.156**	0.014	− 0.022	0.01	− 0.049	− 0.001	− 0.24**	0.014
0.4	− 0.096**	0.009	− 0.161**	0.013	− 0.01	0.004	− 0.062	0.004	− 0.205**	0.006
0.5	− 0.114**	0.007	− 0.103	0.004	0.032	0.003	− 0.093	− 0.001	− 0.206**	0.001
0.6	− 0.077	0.006	− 0.063	0.005	0.043	0.001	− 0.107**	0.002	− 0.272*	− 0.001
0.7	− 0.082	0.004	− 0.165	0.001	0.062*	− 0.001	− 0.011	− 0.003	− 0.288**	0.002
0.8	0.097	− 0.006	− 0.124	− 0.001	0.067**	− 0.006	0.014	− 0.003	− 0.308**	0.004
0.9	− 0.092	− 0.01	− 0.162	− 0.009	0.089	0.023	− 0.004	0.006	− 0.169	0.014

	Japan		Mexico		Russia		S. Africa		S. Arabia	
	COV-19	VAC	COV-19	VAC	COV-19	VAC	COV-19	VAC	COV-19	VAC
0.1	− 0.015	− 0.003	− 0.061	0.007	− 0.249	− 0.006	− 0.106	0.031	− 0.026	0.015*
0.2	− 0.02	− 0.003	− 0.056	0.005	− 0.315	0.018	− 0.081	0.016	− 0.04	0.004
0.3	− 0.008	− 0.008	− 0.082	0.007	− 0.195	0.013	− 0.094	0.018	− 0.024	0.009
0.4	0.003	− 0.009	− 0.138	− 0.001	− 0.147	− 0.012	− 0.051	0.021	− 0.037	0.007
0.5	− 0.002	− 0.009	− 0.197*	− 0.01	− 0.203	− 0.008	− 0.059	0.026	− 0.019	0.003
0.6	0.022	− 0.014*	− 0.187	− 0.007	− 0.229	− 0.01	− 0.109	0.009	0.062	0.005
0.7	0.013	− 0.012	− 0.171	− 0.011	− 0.233	− 0.007	− 0.094	0.013	0.056	0.002
0.8	0.025	− 0.017**	− 0.062	− 0.01	− 0.309	− 0.017	− 0.076	− 0.01	0.043	− 0.001
0.9	0.068*	− 0.016**	− 0.107**	− 0.015	− 0.201	0.011	− 0.052	0.009	0.037	− 0.001

	S. Korea		Turkey		UK		US	
	COV-19	VAC	COV-19	VAC	COV-19	VAC	COV-19	VAC
0.1	− 0.029	0.004	− 0.182	− 0.001	− 0.196***	0.019	− 0.067	− 0.019
0.2	− 0.045*	0.006	0.017	0.004	− 0.183***	0.006	− 0.207*	0.001
0.3	− 0.045*	0.004	− 0.03	0.002	− 0.143	0.001	− 0.219**	0.006
0.4	− 0.013	0.017	− 0.096	− 0.011	− 0.117	0.008	− 0.273***	0.015
0.5	0.008	0.011	− 0.084	− 0.02	− 0.094	0.001	− 0.257***	0.014
0.6	0.004	0.009	− 0.083	− 0.022	0.051	− 0.011	− 0.321***	0.009
0.7	0.01	0.003	0.065	− 0.017	0.042	− 0.014	− 0.242*	0.021
0.8	0.009	0.002	0.05	− 0.021	0.064	− 0.015	− 0.21*	− 0.003
0.9	− 0.009	0.002	0.011	− 0.016	0.069	− 0.006	− 0.233**	− 0.011

***, **, and * indicate statistically significant coefficients at the 1%, 5%, and 10% levels, respectively.

Table 11 presents the results of the country-level quantile regression model for stock market volatility. According to the results in Table 11, negative investor sentiment significantly increases stock market volatility at certain quantiles in all countries, except for Brazil and Turkey. We also find a negative and significant relationship between positive investor sentiment and stock market volatility at least in one quantile in Australia, Canada, France, Germany, India, Indonesia, Italy, Mexico, South Africa, and the United Kingdom.

**Table 11:** Country quantile regression model results for volatility

Dependent variable: LVOL										
	Argentina		Australia		Brazil		Canada		China	
	COV-19	VAC	COV-19	VAC	COV-19	VAC	COV-19	VAC	COV-19	VAC
0.1	0.023	0.001	0.045***	− 0.001	− 0.004	− 0.001	0.045***	− 0.014***	0.022	− 0.002
0.2	0.019	0.002	0.036***	− 0.002	− 0.007	− 0.002	0.049***	− 0.01	0.021	− 0.001
0.3	0.024	0.001	0.032***	− 0.002	0.002	− 0.003	0.054***	− 0.005	0.035***	− 0.003
0.4	0.021	− 0.001	0.029**	− 0.001	0.001	0.002	0.055***	− 0.003	0.027**	− 0.002
0.5	0.027*	− 0.002	0.034***	− 0.002	0.013	0.002	0.044***	− 0.005	0.027**	− 0.001
0.6	0.026*	− 0.001	0.034***	− 0.003	0.021	0.001	0.044***	− 0.004	0.021*	0.002
0.7	0.022	0.003	0.032***	− 0.004	0.031	00.01	0.035***	− 0.002	0.022*	0.002
0.8	0.019	0.001	0.034***	− 0.007**	0.029	− 0.001	0.037***	0.001	0.011	0.002
0.9	0.01	0.002	0.028***	− 0.008***	0.017	− 0.003	0.018	0.002	0.012	0.001

	France		Germany		India		Indonesia		Italy	
	COV-19	VAC	COV-19	VAC	COV-19	VAC	COV-19	VAC	COV-19	VAC
0.1	0.035*	− 0.01*	0.007	− 0.008	0.029***	− 0.001	0.04**	0.001	0.027	− 0.006
0.2	0.028***	− 0.01***	0.004	− 0.01	0.031***	− 0.001	0.051***	− 0.002	0.015	− 0.006
0.3	0.034***	− 0.011***	0.041**	− 0.013***	0.021***	− 0.002	0.026*	0.002	0.007	− 0.003
0.4	0.032***	− 0.008***	0.044***	− 0.011***	0.026***	− 0.003	0.025	0.001	0.003	− 0.006
0.5	0.029**	− 0.008***	0.038**	− 0.011***	0.024**	− 0.003	0.024*	− 0.002	0.011	− 0.002
0.6	0.025**	− 0.008***	0.039***	− 0.011***	0.013	− 0.005*	0.025*	− 0.003	0.023	− 0.006**
0.7	0.026***	− 0.008***	0.042***	− 0.005	0.006	− 0.006**	0.03**	− 0.005	0.047***	− 0.005*
0.8	0.029**	− 0.01***	0.039***	− 0.006*	0.026	− 0.005	0.04***	− 0.007*	0.051***	− 0.006**
0.9	0.047**	− 0.01***	0.016	− 0.008*	0.03	− 0.007*	0.037*	− 0.006	0.037*	− 0.003

	Japan		Mexico		Russia		S. Africa		S. Arabia	
	COV-19	VAC	COV-19	VAC	COV-19	VAC	COV-19	VAC	COV-19	VAC
0.1	0.025***	− 0.005	0.027***	− 0.002	0.05***	0.003	0.023	− 0.001	− 0.001	− 0.002
0.2	0.021***	− 0.002	0.025***	− 0.001	0.04**	0.002	0.020	− 0.001	0.012	0.001
0.3	0.015**	− 0.001	0.018*	− 0.002	0.03*	0.001	0.029**	0.002	0.015***	0.003
0.4	0.013*	− 0.001	0.009	− 0.004	0.032*	− 0.001	0.023**	− 0.001	0.013**	0.002
0.5	0.012*	0.001	0.012	− 0.004	0.037**	0.002	0.019*	− 0.002	0.016**	0.002
0.6	0.009	0.002	0.016	− 0.004	0.034**	0.001	0.024**	− 0.005*	0.01*	0.003
0.7	0.01	0.004	0.016	− 0.003	0.027	0.001	0.019*	− 0.006*	0.007	0.002
0.8	0.003	0.003	0.021	− 0.003	0.061	− 0.001	0.012	− 0.007**	0.013	0.003
0.9	0.022	− 0.002	0.017	− 0.005**	0.068	− 0.001	0.013	− 0.006***	0.008	0.002

	S. Korea		Turkey		UK		US	
	COV-19	VAC	COV-19	VAC	COV-19	VAC	COV-19	VAC
0.1	0.013***	0.01**	0.005	0.004	0.049***	− 0.003	− 0.019	− 0.009
0.2	0.014***	0.007	0.02	0.001	0.029**	− 0.007**	0.013	− 0.006
0.3	0.013***	0.006	− 0.01	0.001	0.027**	− 0.003	0.037	− 0.005
0.4	0.012***	0.006	0.01	− 0.001	0.02	− 0.004	0.031	− 0.002
0.5	0.011***	0.005	0.009	− 0.001	0.036**	− 0.006	0.027	− 0.001
0.6	0.01**	0.005	0.002	− 0.002	0.039**	− 0.005	0.042**	0.001
0.7	0.009**	0.006**	− 0.001	− 0.003	0.037**	− 0.005	0.031**	− 0.001
0.8	0.03	0.004	− 0.001	− 0.003	0.04**	− 0.006	0.036***	− 0.003
0.9	0.039*	0.002	0.01	0.004	0.021	− 0.01***	0.035	− 0.003

***, **, and * indicate statistically significant coefficients at the 1%, 5%, and 10% levels, respectively.

Overall, the country-specific regression results are consistent with the panel data results, where the effect of negative and positive investor sentiments on stock market volatility is stronger than the effect on stock market returns. Therefore, stock market volatility seems to be sensitive to the Google search volume related to COVID-19 and COVID-19 vaccines; this result is consistent with the empirical findings of Ambros et al. (2021). In addition, developed stock markets are more affected by investor sentiment than emerging stock markets because the estimated coefficients for investor sentiment tend to be more significant for developed countries. This is similar to the empirical findings in the literature. For instance, Smales (2021) found that the stock market returns of G7 countries are more affected by investor sentiment than the stock market returns of emerging countries. Rouatbi et al. (2021) examined the impact of vaccinations on developed and emerging stock markets and found that an increase in vaccination has more effects on the former than the latter.

## Conclusions

Behavioral finance research suggests that investors’ emotions and anxiety affect their investment decisions in stock markets. In this study, we use GSVI data to construct negative investor sentiment (proxied by COVID-19-related terms) and positive investor sentiment (proxied by COVID-19 vaccine-related terms). We investigate the relationship between positive and negative investor sentiments and G20 stock market returns and volatility by using various methods, including panel regression with fixed effects, quantile regressions, PVAR, and country-level time-series regressions. Using weekly data from March 2020 to May 2021, we find significant relationships between investor sentiment and stock market returns and volatility. Specifically, an increase in positive investor sentiment leads to an increase in stock returns while negative investor sentiment decreases stock returns on the left-hand side of the distribution. The effect of investor sentiment on volatility is consistent across the distribution: negative sentiment increases volatility, whereas positive sentiment reduces volatility. Finally, these results are robust as the Granger causality tests and PVAR model corroborate them.

Our empirical results are consistent with those of Lyocsa et al. (2020), Chundakkadan and Nedumparambil (2021), and Smale (2021). The panel data model results show that the impact of investor sentiment on stock market volatility is stronger than that on stock market returns. Therefore, stock market volatility seems to be more sensitive to the volume of Google search terms related to COVID-19 and COVID-19 vaccines; this result is consistent with the empirical findings of Ambros et al. (2021). The country-level regression results are mostly consistent with the panel data, and the effect of investor sentiment on stock market volatility is stronger than that on stock market returns. In addition, developed stock markets are more affected by investor sentiment than emerging stock markets because the estimated coefficients for investor sentiment are more significant in developed countries. Specifically, the results for European countries, such as Germany, France, Italy, and the United Kingdom stand out as their stock markets are significantly affected by investor sentiment. Although Russia and China are among the countries producing some COVID-19 vaccines, we cannot validate a significant relationship between positive investor sentiment and stock market returns or volatility for these countries based on quantile regressions.

The emergence of new variants of COVID-19 leads to high levels of uncertainty globally. Investor concerns about COVID-19 seem to have a negative impact on financial markets. However, developments and news about COVID-19 vaccines seem to be a good proxy for positive investor sentiment, which has a positive impact on financial markets. Although COVID-19 first emerged in China, we cannot find significant investor sentiment on Chinese stock market returns based on regression and quantile regression models. We also find that positive investor sentiment significantly reduces stock market return volatility in Germany and the United Kingdom, both of which produce COVID-19 vaccines.

The Google search volume for COVID-19 terms negatively affects stock markets during the ongoing COVID-19. Meanwhile, the Google search for COVID-19 vaccine-related terms and the prospect of an end to the pandemic positively affect stock markets in G20 countries. Thus, Google search terms seem to be good proxies for investor sentiments. The findings may have portfolio implications as the proxies for positive and negative investor sentiments seem to be good predictors of stock returns and volatility during the pandemic. Moreover, it is known that a lack of clarity from public health authorities on vaccine safety has allowed some false claims on the efficacy of vaccines and some conspiracy theories to take hold. Our results suggest the need to formulate a health policy that communicates rapid and accurate information about COVID-19 to mitigate the effects of the pandemic on financial markets. Finally, authorities should adopt policies that convey realistic data on the effects of vaccines on the efforts to end the pandemic. This is particularly important as there is some evidence that government policy response to the pandemic has a moderating role in the relationship between investor sentiment and stock returns.

Future research can extend the analysis by using more countries and different investor sentiment indices. In addition, the sample can be extended to include more data as the global pandemic unfolds, and econometric analyses that rely on high-frequency data can be used. Such analysis can be expected to yield more robust results.

## Appendix

See Appendix Tables

**Table 12:** Country regression model results

Countries	Constant	COV-19	VAC	CASES	FX	GOLD	BOND	R^2^	F-stat
Dependent variable: CAR									
Argentina	75.538*	− 0.091	0.014	3.159	1.085	− 0.705	− 19.909*	0.084	0.865
Australia	3.622**	− 0.098***	− 0.004	− 0.487	1.344***	0.201	− 0.864	0.372	5.548***
Brazil	− 6.616	− 0.062	0.010	− 1.508	1.760*	− 1.557*	0.671	0.178	2.030*
Canada	2.871	− 0.144***	0.016	2.995	4.698***	− 0.191	1.204	0.439	7.322***
China	− 7.245	0.007	− 0.007	0.536	0.648	− 0.318	2.410	0.080	0.815
France	1.688	− 0.096**	0.003	− 0.208	1.966**	− 0.356	1.736	0.241	2.973**
Germany	5.198	− 0.150**	0.004	4.035*	1.538**	− 0.086	5.144	0.258	3.247***
India	− 14.396	0.043	0.007	− 7.949***	0.177	− 0.328	2.328	0.272	3.487***
Indonesia	− 1.235	− 0.029	− 0.002	− 1.029	− 0.472	− 0.354	0.260	0.276	3.560***
Italy	2.620	− 0.215***	0.003	3.614	2.444**	− 0.355	1.382	0.277	3.578***
Japan	− 0.274	0.018	− 0.010*	− 2.968*	− 1.528***	0.049	18.048	0.311	4.231***
Mexico	− 6.035	− 0.113***	− 0.006	− 1.921	1.204***	− 0.398	1.555	0.330	4.615***
Russia	9.880	− 0.347**	− 0.005	14.188	0.150	− 0.284	− 0.989	0.197	2.291**
S. Africa	− 10.091	− 0.074	0.001	− 0.789	0.728	− 0.470	1.177	0.112	1.187
S. Arabia	− 0.941	0.027	0.006*	− 2.755	− 2.570	− 0.775**	−	0.187	2.623**
S. Korea	2.406	− 0.018	0.003	− 0.272	− 0.789**	− 0.082	− 1.674	0.152	1.684
Turkey	− 7.898	− 0.045	− 0.016	− 0.511	− 0.413	− 1.012***	0.735	0.514	9.887***
UK	0.897	− 0.093**	− 0.001	0.450	1.685***	− 0.140	2.943	0.256	3.214***
US	4.295**	− 0.213***	0.002	3.456*	6.738***	0.431	− 0.458	0.614	17.481***

Dependent variable: LVOL									
Argentina	10.516**	0.023***	0.001	0.501*	0.360	− 0.017	− 2.120*	0.314	4.288***
Australia	1.149**	0.032***	− 0.004*	− 0.101	0.003	− 0.017	0.505	0.512	9.739***
Brazil	4.789***	0.007	− 0.001	1.718***	− 0.028	0.009	− 0.263	0.620	15.255***
Canada	0.362	0.043***	− 0.005	− 0.729	0.057	0.018	− 0.001	0.605	14.342***
China	− 0.689	0.024***	− 0.001	− 0.225**	0.413*	0.030	0.785	0.229	2.787**
France	2.110***	0.031***	− 0.009***	− 0.073	0.045	− 0.024	2.257***	0.570	12.392***
Germany	2.678***	0.035**	− 0.008***	− 0.145	0.079	0.049	2.080	0.377	5.654***
India	− 3.391	0.022***	− 0.003**	1.235*	− 0.073	− 0.059	0.791	0.608	14.483***
Indonesia	1.319	0.027***	− 0.001	0.498	− 0.099	0.019	0.050	0.480	8.633***
Italy	0.485	0.027**	− 0.004*	0.417	− 0.012	− 0.052	1.224***	0.591	13.495***
Japan	1.064***	0.012***	0.001	0.545	− 0.008	− 0.037	− 2.210	0.203	2.382**
Mexico	0.470	0.014**	− 0.003**	0.371	0.016	− 0.005	0.300	0.535	10.760***
Russia	1.778	0.039***	0.001	0.014	0.140***	− 0.083*	0.017	0.536	10.798***
S. Africa	0.031	0.018**	− 0.002	0.305**	0.016	0.016	0.247	0.570	12.387***
S. Arabia	0.347	0.010**	0.001	1.871***	− 6.647*	− 0.147***	−	0.491	11.034***
S. Korea	3.047***	0.015***	0.003	− 0.584**	0.183	− 0.080*	− 0.862*	0.308	4.164***
Turkey	0.965	0.006	− 0.001	0.550***	0.114	− 0.082	0.108	0.316	4.317***
UK	1.598***	0.034***	− 0.005***	0.013	− 0.022	− 0.009	− 0.153	0.477	8.515***
US	1.021	0.029*	− 0.004	0.636	0.126	0.039	0.182	0.477	10.455***

^***^, **, and * indicate statistically significant coefficients at 1%, 5% and 10% significance level, respectively

12 and 13.

**Table 13:** Country quantile regression model results

	Quantiles								
Dependent variable: CAR									
Argentina	**0.1**	**0.2**	**0.3**	**0.4**	**0.5**	**0.6**	**0.7**	**0.8**	**0.9**
Constant	191.522***	172.901**	93.394	86.469	52.564	3.035	− 9.103	11.234	68.382
COV-19	− 0.408	− 0.423	− 0.031	− 0.047	0.06	0.184	0.133	0.069	− 0.112
VAC	0.065***	0.048*	0.04*	0.025	0.015	0.001	− 0.004	− 0.011	− 0.059*
CASES	3.121	11.563	2.281	3.858	0.37	− 4.981	− 4.529	− 1.84	− 2.314
FX	0.77	3.41	3.101	0.416	− 0.461	− 1.713	− 0.711	2.122	0.363
GOLD	0.369	− 0.482	− 0.406	− 0.439	− 0.189	− 0.204	− 0.52	0.054	− 1.178
BOND	− 51.81***	− 46.25**	− 26.679	− 23.649	− 14.691	− 1.105	2.623	− 2.328	− 13.093
**Australia**	**0.1**	**0.2**	**0.3**	**0.4**	**0.5**	**0.6**	**0.7**	**0.8**	**0.9**
Constant	− 1.44	− 0.06	0.497	− 0.199	4.019	6.071*	4.504	4.981	10.888
COV-19	− 0.135***	− 0.138***	− 0.101	− 0.046	− 0.085***	− 0.091***	− 0.096***	− 0.086*	− 0.016
VAC	− 0.003	− 0.001	0.002	0.001	− 0.007	0.003	− 0.006	− 0.023*	0.02
CASES	0.339	0.333	− 0.279	− 0.5	− 0.058	0.531	1.166	0.133	− 0.191
FX	0.7***	0.737***	1.345	1.241**	1.898***	1.856***	1.522**	1.145	1.799
GOLD	0.14	0.036	0.091	0.08	0.115	0.18	0.237	0.62*	− 0.171
BOND	0.961	0.12	− 0.733	− 0.38	− 1.294	− 3.346	− 0.249	2.084	− 6.504
**Brazil**	**0.1**	**0.2**	**0.3**	**0.4**	**0.5**	**0.6**	**0.7**	**0.8**	**0.9**
Constant	− 24.72	− 5.049	− 11.433	− 10.65	− 16.407	− 5.821	− 1.527	3.215	21.897
COV-19	− 0.092	− 0.056	0.017	0.086	0.015	− 0.035	0.052	− 0.097	− 0.376
VAC	0.029	0.044	0.03	0.021	0.002	− 0.01	− 0.013	− 0.007	0.027
CASES	− 1.798	− 8.055	− 10.72	− 4.964	− 3.486	− 2.112	− 3.212	3.083	17.554
FX	2.69***	1.336	1.647	1.244	1.053	0.505	0.32	0.21	2.375
GOLD	− 2.045***	− 1.478	− 1.633	− 1.214	− 1.033	− 0.933	− 0.789	− 0.48	− 0.616
BOND	1.118	− 1.042	0.17	0.271	1.865	1.22	0.661	0.663	− 1.186
**Canada**	**0.1**	**0.2**	**0.3**	**0.4**	**0.5**	**0.6**	**0.7**	**0.8**	**0.9**
Constant	4.385	3.077	1.414	2.157	3.731	3.328	1.547	2.835	3.288
COV-19	− 0.244***	− 0.222***	− 0.183*	− 0.187*	− 0.165**	− 0.137	− 0.07	− 0.086	0.022
VAC	0.054*	0.047*	0.019	0.023	0.012	0.005	− 0.008	− 0.011	0.017
CASES	7.135	3.552	1.79	3.841	0.137	− 0.08	1.581	1.187	2.445
FX	5.959***	5.598***	4.411***	4.502***	4.035***	5.183**	4.876**	5.27***	5.712***
GOLD	0.316	− 0.359	− 0.036	− 0.251	− 0.036	− 0.148	− 0.106	0.126	− 0.334
BOND	− 7.083	− 3.398	0.97	1.421	1.77	2.692	5.494	7.202	1.436
**China**	**0.1**	**0.2**	**0.3**	**0.4**	**0.5**	**0.6**	**0.7**	**0.8**	**0.9**
Constant	− 34.813*	− 6.303	− 15.386	− 15.3	− 8.52	− 12.213	6.356	− 0.491	− 14.671
COV-19	0.038	− 0.068	0.056	0.078	0.047	0.08	0.026	0.045	0.083
VAC	− 0.002	− 0.003	− 0.008	− 0.01	− 0.011	− 0.008	− 0.001	− 0.003	− 0.009
CASES	− 2.611	− 0.952	− 0.017	0.595	0.349	0.097	1.083	0.995	0.858
FX	1.592	− 0.399	− 0.228	− 1.077	− 0.927	− 0.922	0.025	1.403	2.029
GOLD	− 0.108	− 0.106	− 0.148	− 0.073	− 0.092	0.001	− 0.093	− 0.297	0.323
BOND	9.327	1.432	4.256	4.552	2.68	3.766	− 1.814	0.791	5.927
**France**	**0.1**	**0.2**	**0.3**	**0.4**	**0.5**	**0.6**	**0.7**	**0.8**	**0.9**
Constant	− 0.842	− 1.815	− 1.111	0.261	1.328	0.981	1.984	2.000	9.208**
COV-19	− 0.252	− 0.125*	− 0.122	− 0.096**	− 0.114**	− 0.077	− 0.082	0.097	− 0.092
VAC	0.028***	0.018	0.018	0.009	0.007	0.006	0.004	− 0.006	− 0.01
CASES	2.809	0.417	0.309	− 2.384*	− 1.775	− 1.137	− 1.309	− 0.301	2.354
FX	0.443	0.663	0.931	1.178*	1.408*	1.47*	1.382*	1.951*	2.887***
GOLD	− 0.104	− 0.443	− 0.267	− 0.243	− 0.09	− 0.094	− 0.387	− 0.368	− 0.794
BOND	4.287	3.195	2.79	0.265	− 1.66	− 1.03	0.385	6.793	5.043
**Germany**	**0.1**	**0.2**	**0.3**	**0.4**	**0.5**	**0.6**	**0.7**	**0.8**	**0.9**
Constant	− 2.246	− 0.316	2.209	2.971	2.881	1.825	7.051	7.688*	15.107**
COV-19	− 0.196***	− 0.209***	− 0.156**	− 0.161**	− 0.103	− 0.063	− 0.165	− 0.124	− 0.162
VAC	0.027**	0.025*	0.014	0.013	0.004	0.005	0.001	− 0.001	− 0.009
CASES	5.587*	5.873	1.514	1.644	1.071	1.688	4.538	6.791**	4.899**
FX	0.362	0.536	0.842	0.946	1.818**	1.428	1.631	1.833*	1.478**
GOLD	− 0.06	− 0.003	− 0.026	0.004	0.227	0.271	0.21	0.019	− 1.204*
BOND	− 0.716	1.302	4.232	5.091	1.424	0.998	2.849	4.416	11.314
**India**	**0.1**	**0.2**	**0.3**	**0.4**	**0.5**	**0.6**	**0.7**	**0.8**	**0.9**
Constant	− 67.846*	− 28.661	− 38.402	− 17.934	− 33.535	− 12.096	− 17.601	2.247	76.611
COV-19	− 0.029	− 0.014	− 0.022	− 0.01	0.032	0.043	0.062*	0.067**	0.089
VAC	0.013	0.017	0.01	0.004	0.003	0.001	− 0.001	− 0.006	0.023
CASES	− 7.492**	− 6.463**	− 5.248**	− 4.251	− 7.555*	− 6.155	− 6.981*	− 7.799*	− 7.002
FX	− 1.057	− 0.665	− 0.045	− 0.259	0.270	− 0.259	0.037	0.021	0.475
GOLD	− 0.314	− 0.169	0.022	0.091	− 0.037	− 0.127	− 0.414	− 0.469	− 0.495
BOND	10.753	4.414	6.253	2.863	5.578	2.047	3.062	− 0.055	− 12.246
**Indonesia**	**0.1**	**0.2**	**0.3**	**0.4**	**0.5**	**0.6**	**0.7**	**0.8**	**0.9**
Constant	10.971	3.297	− 2.204	− 5.358	− 13.429	− 14.247	− 6.952	− 6.321	− 7.409
COV-19	− 0.07	− 0.026	− 0.049	− 0.062	− 0.093	− 0.107**	− 0.011	0.014	− 0.004
VAC	− 0.004	− 0.004	− 0.001	0.004	− 0.001	0.002	− 0.003	− 0.003	0.006
CASES	0.87	− 0.124	0.35	0.62	1.195	1.354	− 2.345	− 3.625	− 2.244
FX	0.134	− 0.567**	− 0.586*	− 0.599*	− 0.718**	− 0.596**	− 0.237	− 0.458	− 0.744
GOLD	− 0.518*	− 0.428	− 0.336	− 0.281	− 0.112	− 0.046	− 0.031	− 0.41	− 0.518**
BOND	− 1.92	− 0.735	0.203	0.731	2.201	2.402	1.237	1.279	1.599
**Italy**	**0.1**	**0.2**	**0.3**	**0.4**	**0.5**	**0.6**	**0.7**	**0.8**	**0.9**
Constant	0.764	0.66	1.263	2.326	2.994	3.883	4.417	5.506	2.434
COV-19	− 0.364***	− 0.264***	− 0.24**	− 0.205**	− 0.206**	− 0.272*	− 0.288**	− 0.308**	− 0.169
VAC	0.006	0.021*	0.014	0.006	0.001	− 0.001	0.002	0.004	0.014
CASES	5.03*	2.836	1.383	− 2.08	− 1.936	2.282	5.007	9.506*	8.876
FX	0.79	1.051	0.979	1.791*	2.679***	2.819***	2.656***	3.123***	3.366***
GOLD	− 0.873**	− 0.452	− 0.304	− 0.013	0.169	0.016	− 0.044	− 0.904	− 0.913
BOND	0.891	− 0.102	− 0.155	− 0.354	0.875	2.735	3.494	5.426	6.763**
**Japan**	**0.1**	**0.2**	**0.3**	**0.4**	**0.5**	**0.6**	**0.7**	**0.8**	**0.9**
Constant	− 1.892	− 0.708	− 0.27	− 0.176	0.742	0.946	1.637	1.704	0.939
COV-19	− 0.015	− 0.02	− 0.008	0.003	− 0.002	0.022	0.013	0.025	0.068*
VAC	− 0.003	− 0.003	− 0.008	− 0.009	− 0.009	− 0.014*	− 0.012	− 0.017**	− 0.016**
CASES	− 2.76*	− 3.404***	− 2.474	− 2.609	− 1.732	− 1.433	− 0.908	− 0.634	− 1.651
FX	− 2.164**	− 1.289*	− 1.407**	− 1.335*	− 0.958*	− 0.737	− 0.878*	− 0.851**	− 1.38***
GOLD	− 0.176	0.068	− 0.04	− 0.067	− 0.053	− 0.073	− 0.107	− 0.039	− 0.073
BOND	8.181	6.004	14.968	12.434	4.913	11.452	5.236	14.628	16.771
**Mexico**	**0.1**	**0.2**	**0.3**	**0.4**	**0.5**	**0.6**	**0.7**	**0.8**	**0.9**
Constant	− 20.84	− 15.449	− 7.088	− 5.909	− 5.076	1.009	4.438	9.99	6.389
COV-19	− 0.061	− 0.056	− 0.082	− 0.138	− 0.197*	− 0.187	− 0.171	− 0.062	− 0.107**
VAC	0.007	0.005	0.007	− 0.001	− 0.01	− 0.007	− 0.011	− 0.01	− 0.015
CASES	− 5.428	− 6.691	− 3.264	− 0.971	2.112	3.318	1.208	− 1.571	− 2.646
FX	1.391***	1.185***	1.181***	1.006**	0.889	0.763	1.016*	0.937	1.018
GOLD	− 0.911**	− 0.892*	− 0.617	− 0.225	0.023	− 0.085	− 0.093	− 0.369	− 0.144
BOND	2.877	2.157	1.033	1.387	1.756	0.834	0.472	− 0.63	0.546
**Russia**	**0.1**	**0.2**	**0.3**	**0.4**	**0.5**	**0.6**	**0.7**	**0.8**	**0.9**
Constant	− 12.453	− 4.149	− 2.676	− 6.649	1.659	6.681	6.938	14.407	23.197
COV-19	− 0.249	− 0.315	− 0.195	− 0.147	− 0.203	− 0.229	− 0.233	− 0.309	− 0.201
VAC	− 0.006	0.018	0.013	− 0.012	− 0.008	− 0.01	− 0.007	− 0.017	0.011
CASES	4.055	8.17	1.741	− 1.373	2.328	5.233	14.203	11.443	18.752
FX	− 0.189	− 0.199	− 0.108	0.229	− 0.078	0.264	− 0.248	0.524	0.203
GOLD	− 0.197	− 0.062	− 0.166	− 0.334	− 0.306	− 0.636	− 0.423	− 0.027	0.515
BOND	1.393	0.084	− 0.086	1.333	0.182	− 0.331	− 0.265	− 0.765	− 2.606
**S. Africa**	**0.1**	**0.2**	**0.3**	**0.4**	**0.5**	**0.6**	**0.7**	**0.8**	**0.9**
Constant	− 41.746	5.033	− 2.045	− 17.162	− 42.841	− 34.029	− 34.468	− 10.362	− 4.74
COV-19	− 0.106	− 0.081	− 0.094	− 0.051	− 0.059	− 0.109	− 0.094	− 0.076	− 0.052
VAC	0.031	0.016	0.018	0.021	0.026	0.009	0.013	− 0.01	0.009
CASES	− 2.163	0.18	− 0.354	− 1.167	− 1.024	− 0.126	0.765	1.747	− 5.187
FX	1.299**	0.905	0.451	0.362	− 0.033	0.12	− 0.077	0.229	1.288
GOLD	− 0.864	− 0.694	− 0.577	− 0.671	− 0.175	0.105	0.244	0.216	0.358
BOND	3.629	− 1.021	− 0.03	1.545	4.405	3.883	3.936	1.693	1.434
**S. Arabia**	**0.1**	**0.2**	**0.3**	**0.4**	**0.5**	**0.6**	**0.7**	**0.8**	**0.9**
Constant	− 4.372**	− 1.87	− 1.959	− 0.599	− 0.026	− 0.258	0.415	2.16*	3.048**
COV-19	− 0.026	− 0.04	− 0.024	− 0.037	− 0.019	0.062	0.056	0.043	0.037
VAC	0.015*	0.004	0.009	0.007	0.003	0.005	0.002	− 0.001	− 0.001
CASES	− 5.369	− 1.549	− 2.231	− 2.153	− 2.212	− 0.872	− 0.23	4.208	5.821
FX	− 20.494	7.917	− 3.6	− 2.038	− 28.114	6.78	− 6.117	− 0.62	− 3.544
GOLD	− 0.094	− 0.45	− 0.635**	− 0.525	− 0.394	− 0.872**	− 0.662	− 0.443	− 0.102
BOND	–	–	–	–	–	–	–	–	–
**S. Korea**	**0.1**	**0.2**	**0.3**	**0.4**	**0.5**	**0.6**	**0.7**	**0.8**	**0.9**
Constant	− 4.759	− 0.316	1.15	3.678	1.261	2.372	2.627	3.431	11.626**
COV-19	− 0.029	− 0.045*	− 0.045*	− 0.013	0.008	0.004	0.01	0.009	− 0.009
VAC	0.004	0.006	0.004	0.017	0.011	0.009	0.003	0.002	0.002
CASES	− 0.519	− 1.203	− 0.008	− 0.928	− 0.727	− 0.585	1.247	0.966	− 1.039
FX	− 0.797	− 1.146*	− 1.246**	− 0.451	− 0.938	− 1.141	− 1.067	− 1.313**	− 0.774
GOLD	0.018	0.085	− 0.124	− 0.11	− 0.024	− 0.081	− 0.014	0.002	− 0.184
BOND	0.976	− 1.099	− 1.475	− 3.842	− 1.754	− 1.984	− 1.405	− 1.508	− 5.238*
**Turkey**	**0.1**	**0.2**	**0.3**	**0.4**	**0.5**	**0.6**	**0.7**	**0.8**	**0.9**
Constant	− 12.015	− 12.473	− 6.301	− 7.107	− 12.359	− 10.402	− 8.796	− 4.036	4.14
COV-19	− 0.182	0.017	− 0.03	− 0.096	− 0.084	− 0.083	0.065	0.05	0.011
VAC	− 0.001	0.004	0.002	− 0.011	− 0.02	− 0.022	− 0.017	− 0.021	− 0.016
CASES	0.749	− 2.095	− 1.948	0.568	− 0.048	− 0.653	− 1.364	− 1.097	− 0.434
FX	− 1.237*	− 0.568	− 0.492	− 0.07	− 0.128	− 0.104	− 0.273	− 0.624	− 0.894
GOLD	− 0.769	− 1.077***	− 1.034***	− 1.281***	− 1.217***	− 1.192**	− 0.883	− 0.498	− 0.184
BOND	0.807	0.657	0.371	0.672	1.146	1.049	0.803	0.599	0.115
**UK**	**0.1**	**0.2**	**0.3**	**0.4**	**0.5**	**0.6**	**0.7**	**0.8**	**0.9**
Constant	− 3.26	− 1.183	− 0.697	− 0.802	0.524	− 1.609	0.18	2.134	3.6
COV-19	− 0.196***	− 0.183***	− 0.143	− 0.117	− 0.094	0.051	0.042	0.064	0.069
VAC	0.019	0.006	0.001	0.008	0.001	− 0.011	− 0.014	− 0.015	− 0.006
CASES	1.574	0.666	2.629	3.141	0.949	0.523	− 1.757	− 5.51	− 8.402*
FX	2.03**	1.528	1.067	1.408	1.48	1.212	1.42**	1.733***	2.024***
GOLD	0.08	− 0.224	− 0.122	− 0.092	0.023	− 0.229	0.028	− 0.264	− 0.536
BOND	2.852	4.374	4.687	3.204	2.164	3.865	3.853	1.925	0.801
**US**	**0.1**	**0.2**	**0.3**	**0.4**	**0.5**	**0.6**	**0.7**	**0.8**	**0.9**
Constant	− 12.032**	0.07	0.749	6.193*	6.154*	8.686**	8.888*	10.221**	12.635***
COV-19	− 0.067	− 0.207*	− 0.219**	− 0.273***	− 0.257***	− 0.321***	− 0.242*	− 0.21*	− 0.233**
VAC	− 0.019	0.001	0.006	0.015	0.014	0.009	0.021	− 0.003	− 0.011
CASES	− 1.213	2.778	4.584	5.723*	5.255	7.124**	5.287	0.067	0.351
FX	6.899***	5.937***	6.547***	7.053***	6.565***	6.598***	6.058***	7.9***	7.634***
GOLD	0.264	0.527	0.335	0.39	0.174	0.202	0.374	0.572	0.418
BOND	7.879**	0.824	0.924	− 2.246	− 2.087	− 2.231	− 3.666	− 1.773	− 1.907

Dependent variable: LVOL									
**Argentina**	**0.1**	**0.2**	**0.3**	**0.4**	**0.5**	**0.6**	**0.7**	**0.8**	**0.9**
Constant	13.219	12.991	10.776	10.494	7.177	3.632	7.439	15.032*	14.703*
COV-19	0.023	0.019	0.024	0.021	0.027*	0.026*	0.022	0.019	0.01
VAC	0.001	0.002	0.001	− 0.001	− 0.002	− 0.001	0.003	0.001	0.002
CASES	0.721	0.858	0.587	0.662	0.316	0.395	0.358	0.466	0.519
FX	1.461	1.051	0.117	− 0.101	− 0.308	− 0.079	0.447	0.41	0.664
GOLD	− 0.118	− 0.066	0.009	0.024	0.02	− 0.003	− 0.022	0.023	0.042
BOND	− 3.323	− 3.124	− 2.254	− 2.078	− 1.106	− 0.169	− 1.215	− 3.066	− 2.892
**Australia**	**0.1**	**0.2**	**0.3**	**0.4**	**0.5**	**0.6**	**0.7**	**0.8**	**0.9**
Constant	− 0.875	0.644	1.533**	1.996***	1.732***	1.859***	2.21***	1.382*	1.739**
COV-19	0.045***	0.036***	0.032***	0.029**	0.034***	0.034***	0.032***	0.034***	0.028***
VAC	− 0.001	− 0.002	− 0.002	− 0.001	− 0.002	− 0.003	− 0.004	− 0.007**	− 0.008***
CASES	− 0.133	− 0.378	− 0.035	− 0.014	0.066	− 0.102	0.022	− 0.312	− 0.204
FX	− 0.024	0.108	0.046	0.077	0.001	− 0.001	− 0.002	− 0.063	− 0.051
GOLD	− 0.082	− 0.002	0.03	0.013	− 0.001	− 0.031	− 0.081	0.048	0.078
BOND	0.64	0.155	− 0.344	− 0.625	− 0.284	− 0.159	− 0.219	1.209	1.375*
**Brazil**	**0.1**	**0.2**	**0.3**	**0.4**	**0.5**	**0.6**	**0.7**	**0.8**	**0.9**
Constant	5.513***	4.117**	4.687***	5.162***	5.544***	5.627***	6.145***	4.632***	3.432
COV-19	− 0.004	− 0.007	0.002	0.001	0.013	0.021	0.031	0.029	0.017
VAC	− 0.001	− 0.002	− 0.003	0.002	0.002	0.001	0.001	− 0.001	− 0.003
CASES	2.64***	2.34***	1.88***	2.25***	2.046**	1.39*	1.021	0.857	0.712
FX	− 0.121	− 0.085	− 0.063	− 0.029	0.035	0.023	0.004	− 0.033	− 0.03
GOLD	0.09**	0.025	0.02	− 0.01	− 0.038	− 0.024	− 0.011	0.002	0.01
BOND	− 0.476*	− 0.198	− 0.262	− 0.378	− 0.422	− 0.409	− 0.466	− 0.227	0.065
**Canada**	**0.1**	**0.2**	**0.3**	**0.4**	**0.5**	**0.6**	**0.7**	**0.8**	**0.9**
Constant	− 1.857**	− 1.042	− 0.29	0.1	0.808	0.843	1.494***	1.809***	3.505***
COV-19	0.045***	0.049***	0.054***	0.055***	0.044***	0.044***	0.035***	0.037***	0.018
VAC	− 0.014***	− 0.01	− 0.005	− 0.003	− 0.005	− 0.004	− 0.002	0.001	0.002
CASES	− 0.114	− 0.893	− 1.751*	− 2.077**	− 1.348	− 1.096	− 0.346	− 0.097	0.858
FX	− 0.222	− 0.043	0.07	0.116	0.109	0.113	0.026	− 0.141	− 0.029
GOLD	− 0.036	− 0.004	0.003	− 0.023	− 0.035	− 0.046	− 0.002	0.026	− 0.036
BOND	1.877**	0.912	− 0.229	− 0.651	− 0.564	− 0.422	− 0.79	− 1.161	− 1.902**
**China**	**0.1**	**0.2**	**0.3**	**0.4**	**0.5**	**0.6**	**0.7**	**0.8**	**0.9**
Constant	− 1.211	− 0.877	− 4.122	− 0.958	− 1.07	0.165	− 0.041	0.543	0.456
COV-19	0.022	0.021	0.035***	0.027**	0.027**	0.021*	0.022*	0.011	0.012
VAC	− 0.002	− 0.001	− 0.003	− 0.002	− 0.001	0.002	0.002	0.002	0.001
CASES	− 0.302*	− 0.216	− 0.372**	− 0.291	− 0.287	− 0.203	− 0.181	− 0.097	− 0.285
FX	0.287	0.312	0.518	0.534	0.468	0.537*	0.386	0.852***	0.688**
GOLD	0.026	0.036	0.08	0.056	0.07	0.059	0.05	0.045	0.033
BOND	0.734	0.639	1.82*	0.847	0.891	0.519	0.635	0.627	0.8
**France**	**0.1**	**0.2**	**0.3**	**0.4**	**0.5**	**0.6**	**0.7**	**0.8**	**0.9**
Constant	1.113	1.823***	1.751***	1.949***	2.179***	2.413***	2.584***	2.905***	2.596***
COV-19	0.035*	0.028***	0.034***	0.032***	0.029**	0.025**	0.026***	0.029**	0.047**
VAC	− 0.01*	− 0.01***	− 0.011***	− 0.008***	− 0.008***	− 0.008***	− 0.008***	− 0.01***	− 0.01***
CASES	− 0.359	− 0.192	− 0.124	0.016	0.159	0.099	0.156	− 0.181	− 0.389
FX	0.021	0.147	0.061	0.012	0.005	0.153	− 0.017	− 0.086	− 0.123
GOLD	0.001	− 0.098	− 0.077	− 0.043	− 0.008	− 0.002	− 0.02	− 0.052	− 0.098
BOND	3.67	3.716***	2.334*	2.692**	2.876**	2.11*	1.349	1.28	0.868
**Germany**	**0.1**	**0.2**	**0.3**	**0.4**	**0.5**	**0.6**	**0.7**	**0.8**	**0.9**
Constant	2.595	3.504**	3.12***	2.667***	3.045***	2.698***	1.495	2.109**	4.474**
COV-19	0.007	0.004	0.041**	0.044***	0.038**	0.039***	0.042***	0.039***	0.016
VAC	− 0.008	− 0.01	− 0.013***	− 0.011***	− 0.011***	− 0.011***	− 0.005	− 0.006*	− 0.008*
CASES	0.562	0.163	− 0.575	− 0.391	− 0.506	− 0.627	− 0.263	− 0.092	0.749
FX	0.449**	0.368*	0.08	0.022	0.073	0.124	0.037	− 0.048	− 0.001
GOLD	0.078	0.054	0.012	0.001	− 0.041	− 0.034	0.054	0.08	0.051
BOND	2.804	3.704	3.362**	2.527	2.544	1.612	− 0.874	− 0.265	2.332
**India**	**0.1**	**0.2**	**0.3**	**0.4**	**0.5**	**0.6**	**0.7**	**0.8**	**0.9**
Constant	3.21	6.086	2.15	5.464	4.136	− 3.653	− 9.138	− 17.155	− 20.861*
COV-19	0.029***	0.031***	0.021***	0.026***	0.024**	0.013	0.006	0.026	0.03
VAC	− 0.001	− 0.001	− 0.002	− 0.003	− 0.003	− 0.005*	− 0.006**	− 0.005	− 0.007*
CASES	1.757***	1.251	1.85***	1.875**	1.918**	1.51	1.564	0.299	− 0.038
FX	− 0.32***	− 0.25	− 0.288*	− 0.207	− 0.23	− 0.134	− 0.082	0.131	0.085
GOLD	− 0.044	− 0.081	− 0.069	− 0.048	− 0.038	− 0.048	− 0.043	0.015	− 0.033
BOND	− 0.504	− 0.942	− 0.227	− 0.768	− 0.53	0.898	1.876	3.237	3.904**
**Indonesia**	**0.1**	**0.2**	**0.3**	**0.4**	**0.5**	**0.6**	**0.7**	**0.8**	**0.9**
Constant	− 0.553	4.386	− 1.145	− 1.643	0.287	2.416	4.909	7.259**	8.818**
COV-19	0.04**	0.051***	0.026*	0.025	0.024*	0.025*	0.03**	0.04***	0.037*
VAC	0.001	− 0.002	0.002	0.001	− 0.002	− 0.003	− 0.005	− 0.007*	− 0.006
CASES	0.616	0.062	0.313	0.167	0.185	− 0.016	− 0.161	− 0.374	0.313
FX	− 0.111	− 0.095	− 0.033	0.019	− 0.004	0.034	0.011	− 0.084	− 0.142
GOLD	0.14	0.05	− 0.069	− 0.055	0.001	− 0.045	− 0.031	0.009	− 0.04
BOND	0.034	− 0.621	0.328	0.453	0.232	− 0.029	− 0.357	− 0.681	− 0.867
**Italy**	**0.1**	**0.2**	**0.3**	**0.4**	**0.5**	**0.6**	**0.7**	**0.8**	**0.9**
Constant	− 0.95	− 0.336	− 0.161	0.402	0.483	1.37**	1.213**	1.675***	1.811***
COV-19	0.027	0.015	0.007	0.003	0.011	0.023	0.047***	0.051***	0.037*
VAC	− 0.006	− 0.006	− 0.003	− 0.006	− 0.002	− 0.006**	− 0.005*	− 0.006**	− 0.003
CASES	0.573	0.366	0.616	0.255	0.111	0.287	0.016	− 0.143	0.076
FX	− 0.143	− 0.066	0.021	0.107	0.053	0.137	− 0.008	0.009	− 0.02
GOLD	− 0.012	− 0.086	− 0.08	− 0.036	− 0.022	− 0.097	− 0.014	− 0.015	− 0.045
BOND	1.709***	1.686***	1.688***	1.661***	1.466***	0.826	0.589	0.228	0.661
**Japan**	**0.1**	**0.2**	**0.3**	**0.4**	**0.5**	**0.6**	**0.7**	**0.8**	**0.9**
Constant	0.024	0.17	0.776*	1.068**	1.156***	1.46***	1.326***	2.029***	1.78***
COV-19	0.025***	0.021***	0.015**	0.013*	0.012*	0.009	0.01	0.003	0.022
VAC	− 0.005	− 0.002	− 0.001	− 0.001	0.001	0.002	0.004	0.003	− 0.002
CASES	− 0.018	0.277	0.386	0.35	0.348	0.288	0.302	1.037**	1.164*
FX	− 0.012	− 0.051	0.031	− 0.004	− 0.014	− 0.004	0.008	− 0.043	− 0.044
GOLD	− 0.141	− 0.079	0.032	0.034	0.025	− 0.013	− 0.014	− 0.106	− 0.058
BOND	− 3.177	2.467	− 1.082	− 1.573	− 1.932	− 5.171	− 5.586	− 7.415	5.582
**Mexico**	**0.1**	**0.2**	**0.3**	**0.4**	**0.5**	**0.6**	**0.7**	**0.8**	**0.9**
Constant	2.214	3.231*	1.61	0.495	1.15	0.715	− 0.819	0.477	− 0.968
COV-19	0.027***	0.025***	0.018*	0.009	0.012	0.016	0.016	0.021	0.017
VAC	− 0.002	− 0.001	− 0.002	− 0.004	− 0.004	− 0.004	− 0.003	− 0.003	− 0.005**
CASES	0.452	0.494	0.309	0.151	0.337	0.256	0.179	0.023	− 0.387
FX	0.062	0.04	0.055	0.049	− 0.001	− 0.021	0.054	0.069	0.004
GOLD	− 0.059	− 0.007	− 0.022	0.01	− 0.032	− 0.04	− 0.077	− 0.081	0.045
BOND	− 0.22	− 0.345	0.005	0.313	0.217	0.278	0.57*	0.352*	0.712*
**Russia**	**0.1**	**0.2**	**0.3**	**0.4**	**0.5**	**0.6**	**0.7**	**0.8**	**0.9**
Constant	2.907	1.929	1.531	0.028	1.907	1.636	2.884	2.351	1.251
COV-19	0.05***	0.04**	0.03*	0.032*	0.037**	0.034**	0.027	0.061	0.068
VAC	0.003	0.002	0.001	− 0.001	0.002	0.001	0.001	− 0.001	− 0.001
CASES	0.356	0.349	0.475	0.037	0.028	0.083	0.599	− 0.71	− 1.139
FX	0.221**	0.176**	0.127	0.131**	0.174***	0.142**	0.127*	0.086	0.082
GOLD	− 0.118*	− 0.076	− 0.039	− 0.052	− 0.077	− 0.095	− 0.12*	− 0.11	− 0.094
BOND	− 0.379	− 0.128	0.028	0.317	− 0.018	0.067	− 0.081	0.014	0.191
**S. Africa**	**0.1**	**0.2**	**0.3**	**0.4**	**0.5**	**0.6**	**0.7**	**0.8**	**0.9**
Constant	− 1.141	0.331	1.825	0.703	0.913	3.478	2.587	− 0.205	0.145
COV-19	0.023	0.02	0.029**	0.023**	0.019*	0.024**	0.019*	0.012	0.013
VAC	− 0.001	− 0.001	0.002	− 0.001	− 0.002	− 0.005*	− 0.006*	− 0.007**	− 0.006***
CASES	0.094	0.386	0.203	0.284	0.333	0.221	0.413*	0.349	0.479
FX	− 0.016	0.079	0.024	0.023	0.029	0.021	− 0.042	0.03	0.079
GOLD	0.031	0.016	0.004	− 0.028	− 0.016	0.003	0.081	0.022	− 0.01
BOND	0.276	0.131	− 0.046	0.137	0.145	− 0.094	0.037	0.376	0.352
**S. Arabia**	**0.1**	**0.2**	**0.3**	**0.4**	**0.5**	**0.6**	**0.7**	**0.8**	**0.9**
Constant	− 0.383	− 0.515	− 0.493	0.067	0.281	0.657*	0.969***	1.089***	1.575***
COV-19	− 0.001	0.012	0.015***	0.013**	0.016**	0.01*	0.007	0.013	0.008
VAC	− 0.002	0.001	0.003	0.002	0.002	0.003	0.002	0.003	0.002
CASES	1.919*	1.548***	1.558***	1.365***	1.457***	1.803**	2.022*	1.567*	2.048*
FX	− 1.032	3.587	− 1.241	− 1.44	− 5.871	− 5.839	− 5.939	− 9.662*	− 9.313*
GOLD	− 0.175	− 0.173**	− 0.149*	− 0.175*	− 0.194**	− 0.137*	− 0.102	− 0.151*	− 0.222**
BOND	–	–	–	–	–	–	–	–	–
**S. Korea**	**0.1**	**0.2**	**0.3**	**0.4**	**0.5**	**0.6**	**0.7**	**0.8**	**0.9**
Constant	3.318***	3.267***	3.338***	3.302***	3.354***	2.96***	2.513**	2.075	3.663*
COV-19	0.013***	0.014***	0.013***	0.012***	0.011***	0.01**	0.009**	0.03	0.039*
VAC	0.01**	0.007	0.006	0.006	0.005	0.005	0.006**	0.004	0.002
CASES	− 1.313**	− 0.858**	− 0.574*	− 0.531	− 0.497	− 0.613*	− 0.578*	0.035	− 0.581
FX	0.356**	0.418**	0.376**	0.357*	0.351*	0.386**	0.358**	0.305	0.26
GOLD	− 0.133***	− 0.14***	− 0.111**	− 0.106*	− 0.107*	− 0.085	− 0.076	− 0.001	0.054
BOND	− 1.888***	− 1.607**	− 1.436**	− 1.313*	− 1.236	− 0.858	− 0.475	− 0.183	− 0.833
**Turkey**	**0.1**	**0.2**	**0.3**	**0.4**	**0.5**	**0.6**	**0.7**	**0.8**	**0.9**
Constant	1.795	− 0.572	1.734	0.692	1.017	1.299	1.752	2.405*	0.383
COV-19	0.005	0.02	− 0.01	0.01	0.009	0.002	− 0.001	− 0.001	0.01
VAC	0.004	0.001	0.001	− 0.001	− 0.001	− 0.002	− 0.003	− 0.003	0.004
CASES	0.349	0.158	0.498	0.459	0.575	0.876**	0.82**	0.714	0.387
FX	0.344	0.155	0.206	0.098	0.094	0.103	0.133*	0.092	0.043
GOLD	− 0.204**	− 0.231***	− 0.164	− 0.12	− 0.094	− 0.061	− 0.049	− 0.039	0.01
BOND	− 0.047	0.155	0.049	0.116	0.107	0.111	0.102	0.071	0.202
**UK**	**0.1**	**0.2**	**0.3**	**0.4**	**0.5**	**0.6**	**0.7**	**0.8**	**0.9**
Constant	− 0.361	1.18**	1.495***	1.894***	1.723***	1.725***	1.869***	1.987***	2.762***
COV-19	0.049***	0.029**	0.027**	0.02	0.036**	0.039**	0.037**	0.04**	0.021
VAC	− 0.003	− 0.007**	− 0.003	− 0.004	− 0.006	− 0.005	− 0.005	− 0.006	− 0.01***
CASES	0.235	− 0.026	0.212	0.344	− 0.341	− 0.139	0.133	− 0.172	0.358
FX	− 0.118	0.02	0.102	0.073	0.118	− 0.034	− 0.114	− 0.12	− 0.078
GOLD	0.309*	− 0.005	− 0.076	− 0.055	− 0.054	− 0.037	− 0.031	0.008	0.02
BOND	− 0.164	0.082	− 0.856	− 0.632	− 0.173	0.013	0.071	0.426	1.227
**US**	**0.1**	**0.2**	**0.3**	**0.4**	**0.5**	**0.6**	**0.7**	**0.8**	**0.9**
Constant	0.575	0.488	0.262	0.651	1.435	1.373*	2.224***	2.049**	2.426*
COV-19	− 0.019	0.013	0.037	0.031	0.027	0.042**	0.031**	0.036***	0.035
VAC	− 0.009	− 0.006	− 0.005	− 0.002	− 0.001	0.001	− 0.001	− 0.003	− 0.003
CASES	3.11	1.671	0.588	0.607	0.879	0.188	0.321	0.049	− 0.074
FX	0.051	0.096	0.025	0.145	0.082	0.071	− 0.007	0.004	0.314
GOLD	0.121	0.109	0.023	0.03	0.064	0.044	− 0.036	0.008	0.07
BOND	0.618	0.391	0.279	− 0.018	− 0.523	− 0.623	− 0.73	− 0.28	− 0.111

***, **, and * indicate statistically significant coefficients at 1%, 5% and 10% significance level, respectively. The bold numbers show quantile levels

## Footnotes

1 We present detailed information on the variables in the next section.

2 There are several justifications for using weekly data. First, when we consider daily data, we could not find Google search data for the Covid-19 vaccine terms in some countries. Second, daily data for the stock market may cause a day of the week anomaly. Finally, using weekly data allows us to calculate realized volatility by using daily return data. Otherwise, calculating daily realized volatility requires intraday data which is difficult to obtain. We start the sample from March 2020 because there is no data on Google search volume for the Covid-19 vaccine before March 2020.

3 The foreign exchange rate is measured vis-a-vis the US dollar for all countries except for the US. We use the trade-weighted US dollar index for the US.

4 Gold price is calculated in national currency for all countries.

5 https://ourworldindata.org/covid-cases

6 To examine the potential endogeneity issue between the stock market and Google indices, we also use an instrumental variable panel regression model. We find similar results with the fixed-effect panel regression model.

7 COV, VAC, CAR and VOL are considered as endogenous variables in the PVAR model. We treat CASES, FX, GOLD and Bond as exogenous variables.

8 We also test the stability of the PVAR model with one lag and find that the PVAR model satisfies the stability condition. The test results are available upon request.

9 The impulse-responses analysis is conducted using Cholesky decomposition where the order of variables is important. We order the variables as COV-19 → VAC → CAR (or LVOL) based on Granger causality test results.

10 To save space, we present model results for all variables in an Appendix.

## References

[CR1] Abrigo MRM, Love I (2016). Estimation of panel vector autoregression in Stata. Stand Genomic Sci.

[CR2] Al-Awadhi AM, Alsaifi K, Al-Awadhi A, Alhammadi S (2020). Death and contagious infectious diseases: Impact of the COVID-19 virus on stock market returns. J Behav Exp Financ.

[CR3] Albulescu CT (2021). COVID-19 and the United States financial markets’ volatility. Financ Res Lett.

[CR4] Ambros M, Frenkel M, Huynh TLD, Kilinc M (2021). COVID-19 pandemic news and stock market reaction during the onset of the crisis: evidence from high-frequency data. Appl Econ Lett.

[CR5] Andrei D, Hasler M (2015). Investor attention and stock market volatility. Rev Financ Stud.

[CR6] Aouadi A, Arouri M, Teulon F (2013). Investor attention and stock market activity: evidence from France. Econ Model.

[CR7] Azimli A (2020). The impact of COVID-19 on the degree of dependence and structure of risk-return relationship: a quantile regression approach. Financ Res Lett.

[CR8] Baker SR, Bloom N, Davis SJ, Kost KJ, Sammon MC, Viratyosin T (2020) The unprecedented stock market impact of COVID-19 (No. w26945). National Bureau of Economic Research

[CR9] Bannigidadmath D, Narayan PK, Phan DHB, Gong Q (2021) How stock markets reacted to COVID-19? Evidence from 25 countries. Finance Research Letters 102161 10.1016/j.frl.2021.102161 PMC8856896 35221817

[CR10] Barber BM, Odean T (2001). The internet and the investor. J Econ Perspect.

[CR11] Brown GW, Cliff MT (2004). Investor sentiment and the near-term stock market. J Empir Financ.

[CR12] Canay IA (2011). A simple approach to quantile regression for panel data. Econom J.

[CR13] Cao KH, Li Q, Liu Y, Woo CK (2021). Covid-19’s adverse effects on a stock market index. Appl Econ Lett.

[CR14] Chemmanur TJ, Yan A (2019). Advertising, attention, and stock returns. Q J Financ.

[CR15] Chen C, Liu L, Zhao N (2020). Fear sentiment, uncertainty, and bitcoin price dynamics: the case of COVID-19. Emerg Mark Financ Trade.

[CR16] Chen R, Qian Q, Jin C, Xu M, Song Q (2020). Investor attention on internet financial markets. Financ Res Lett.

[CR17] Chen T (2017). Investor attention and global stock returns. J Behav Financ.

[CR18] Cheng IH (2020). Volatility markets underreacted to the early stages of the COVID-19 pandemic. Rev Asset Pricing Stud.

[CR19] Chundakkadan, R, Nedumparambil, E (2021) In search of COVID-19 and stock market behavior. Glob Financ J 100639 10.1016/j.gfj.2021.100639 PMC9620496 38013956

[CR20] Corbet S, Larkin C, Lucey B (2020). The contagion effects of the COVID-19 pandemic: evidence from gold and cryptocurrencies. Financ Res Lett.

[CR21] Costola M, Iacopini M, Santagiustina CRMA (2021). Google search volumes and the financial markets during the COVID-19 outbreak. Financ Res Lett.

[CR22] Da Z, Engelberg J, Gao P (2011). In search of attention. J Financ.

[CR23] Da Z, Engelberg J, Gao P (2015). The sum of all FEARS investor sentiment and asset prices. Rev Financ Stud.

[CR24] Dergiades T (2012). Do investors’ sentiment dynamics affect stock returns? Evidence from the US economy. Econ Lett.

[CR25] Donadelli M, Kizys R, Riedel M (2017). Dangerous infectious diseases: bad news for main street, good news for Wall Street?. J Financ Markets.

[CR26] Fang X, Jiang Y, Qian Z (2014). The effects of individual investors' attention on stock returns: Evidence from the ChiNext market. Emerg Mark Financ Trade.

[CR27] Gao GP, Moulton PC, Ng DT (2017). Institutional ownership and return predictability across economically unrelated stocks. J Financ Intermed.

[CR28] Gil-Alana LA, Claudio-Quiroga G (2020). The COVID-19 impact on the Asian stock markets. Asian Econ Lett.

[CR29] Goel G, Dash SR (2022) Investor sentiment and government policy interventions: evidence from COVID-19 spread. J Financ Econ Policy 14(2):242–267

[CR30] Gormsen NJ, Koijen RS (2020). Coronavirus: impact on stock prices and growth expectations. Rev Asset Pricing Stud.

[CR31] Han L, Li Z, Yin L (2018). Investor attention and stock returns: international evidence. Emerg Mark Financ Trade.

[CR32] Hansen LP (1982). Large sample properties of generalized method of moments estimators. Econometrica.

[CR33] Harjoto MA, Rossi F, Paglia JK (2021). COVID-19: Stock market reactions to the shock and the stimulus. Appl Econ Lett.

[CR34] Haroon O, Rizvi SAR (2020). COVID-19: Media coverage and financial markets behavior—a sectoral inquiry. J Behav Exp Financ.

[CR35] Haroon O, Rizvi SAR (2020). Flatten the curve and stock market liquidity–an inquiry into emerging economies. Emerg Markets Financ Trade.

[CR36] Hashmi SM, Chang BH, Rong L (2021). Asymmetric effect of COVID-19 pandemic on E7 stock indices: evidence from quantile-on-quantile regression approach. Res Int Bus Financ.

[CR37] Heyman D, Lescrauwaet M, Stieperaere H (2019). Investor attention and short-term return reversals. Financ Res Lett.

[CR38] Hirshleifer D, Lim SS, Teoh SH (2011). Limited investor attention and stock market misreactions to accounting information. Rev Asset Pricing Stud.

[CR39] Hood M, Malik F (2013). Is gold the best hedge and a safe haven under changing stock market volatility?. Rev Financ Econ.

[CR40] Hossain M (2021). The effect of the Covid-19 on sharing economy activities. J Clean Prod.

[CR41] Humpe A, Macmillan P (2009). Can macroeconomic variables explain long-term stock market movements? A comparison of the US and Japan. Appl Financ Econo.

[CR42] Hussain A, Tahir A, Hussain Z, Sheikh Z, Gogate M, Dashtipour K, Sheikh A (2021). Artificial intelligence–enabled analysis of public attitudes on Facebook and Twitter toward Covid-19 vaccines in the United Kingdom and the United States: observational study. J Med Internet Res.

[CR43] Ichev R, Marinč M (2018). Stock prices and geographic proximity of information: Evidence from the Ebola outbreak. Int Rev Financ Anal.

[CR44] Iyke BN, Ho SY (2021). Investor attention on COVID-19 and African stock returns. MethodsX.

[CR45] Jain A, Biswal PC (2016). Dynamic linkages among oil price, gold price, exchange rate, and stock market in India. Resour Policy.

[CR46] Kahneman D, Tversky A (1979). Prospect theory: an analysis of decision under risk. Econometrica.

[CR47] Kamstra MJ, Kramer LA, Levi MD (2003). Winter blues: a SAD stock market cycle. Am Econ Rev.

[CR48] Kaplanski G, Levy H (2010). Sentiment and stock prices: the case of aviation disasters. J Financ Econ.

[CR49] Kim N, Lučivjanská K, Molnár P, Villa R (2019). Google searches and stock market activity: evidence from Norway. Financ Res Lett.

[CR50] Koenker R (2004). Quantile regression for longitudinal data. J Multivar Anal.

[CR51] Koenker R, Bassett G (1978). Regression quantiles. Econometrica.

[CR52] Kou G, Olgu Akdeniz Ö, Dinçer H, Yüksel S (2021). Fintech investments in European banks: a hybrid IT2 fuzzy multidimensional decision-making approach. Financ Innov.

[CR53] Kwok SWH, Vadde SK, Wang G (2021) Twitter speaks: an analysis of Australian Twitter users' topics and sentiments about COVID-19 vaccination using machine learning. J Med Internet Res 23(5):e26953 10.2196/26953 PMC8136408 33886492

[CR54] Levin A, Lin CF, Chu JCS (2002). Unit root tests in panel data: asymptotic and finite-sample properties. J Econom.

[CR55] Li H, Guo Y, Park SY (2017). Asymmetric relationship between investors’ sentiment and stock returns: evidence from a quantile non-causality test. Int Rev Financ.

[CR56] Li T, Kou G, Peng Y, Yu PS (2021). An integrated cluster detection, optimization and interpretation approach for financial data. IEEE Trans Cybern.

[CR57] Liu D, Sun W, Zhang X (2020). Is the Chinese economy well positioned to fight the COVID-19 pandemic? The financial cycle perspective. Emerg Mark Financ Trade.

[CR58] Liu H, Manzoor A, Wang C, Zhang L, Manzoor Z (2020). The COVID-19 outbreak and affected countries stock markets response. Int J Environ Res Public Health.

[CR59] López-Cabarcos MÁ, Piñeiro-Chousa J, Pérez-Pico AM (2017). The impact technical and non-technical investors have on the stock market: evidence from the sentiment extracted from social networks. J Behav Exp Financ.

[CR60] Lyocsa S, Baumöhl E, Výrost T, Molnár P (2020). Fear of the coronavirus and the stock markets. Financ Res Lett.

[CR61] Ma C, Xiao S, Ma Z (2018). Investor sentiment and the prediction of stock returns: a quantile regression approach. Appl Econ.

[CR62] Ma R, Sun B, Zhai P, Jin Y (2021). Hedging stock market risks: Can gold really beat bonds?. Financ Res Lett.

[CR63] Machado JAF, Silva JMCS (2019). Quantiles via moments. J Econom.

[CR64] Mazur M, Dang M, Vega M (2021). COVID-19 and the March 2020 stock market crash. Evidence from S&P1500. Financ Res Lett.

[CR65] Mishra AK, Rath BN, Dash AK (2020). Does the Indian financial market nosedive because of the COVID-19 outbreak, in comparison to after demonetisation and the GST. Emerg Mark Financ Trade.

[CR66] Narayan PK, Iyke BN, Sharma SS (2021). New measures of the COVID-19 pandemic: a new time-series dataset. Asian Econ Lett.

[CR67] Narayan PK, Phan DHB, Liu G (2021). COVID-19 lockdowns, stimulus packages, travel bans, and stock returns. Financ Res Lett.

[CR68] Nofsinger JR (2005). Social mood and financial economics. J Behav Financ.

[CR69] Padhan R, Prabheesh KP (2021). The economics of COVID-19 pandemic: a survey. Econ Anal Policy.

[CR70] Padungsaksawasdi C, Treepongkaruna S, Brooks R (2019). Investor attention and stock market activities: new evidence from panel data. Int J Financ Stud.

[CR71] Pesaran MH (2007). A simple panel unit root test in the presence of cross-section dependence. J Appl Economet.

[CR72] Pesaran MH (2015). Testing weak cross-sectional dependence in large panels. Economet Rev.

[CR73] Phan DHB, Narayan PK (2020). Country responses and the reaction of the stock market to COVID-19—a preliminary exposition. Emerg Mark Financ Trade.

[CR74] Piñeiro-Chousa J, López-Cabarcos MÁ, Quiñoá-Piñeiro L, Pérez-Pico AM (2022). US biopharmaceutical companies’ stock market reaction to the COVID-19 pandemic. Understanding the concept of the ‘paradoxical spiral’ from a sustainability perspective. Technol Forecast Soc Change.

[CR75] Rouatbi W, Demir E, Kizys R, Zaremba A (2021). Immunizing markets against the pandemic: COVID-19 vaccinations and stock volatility around the world. Int Rev Financ Anal.

[CR76] Sattar NS, Arifuzzaman S (2021). Covid-19 vaccination awareness and aftermath: public sentiment analysis on Twitter data and vaccinated population prediction in the USA. Appl Sci.

[CR77] Sharif A, Aloui C, Yarovaya L (2020). COVID-19 pandemic, oil prices, stock market, geopolitical risk and policy uncertainty nexus in the US economy: fresh evidence from the wavelet-based approach. Int Rev Financ Anal.

[CR78] Sharma SS (2020). A note on the Asian market volatility during the COVID-19 pandemic. Asian Econ Lett.

[CR79] Shu HC (2010). Investor mood and financial markets. J Econ Behav Organ.

[CR80] Smales LA (2021). Investor attention and global market returns during the COVID-19 crisis. Int Rev Financ Anal.

[CR81] Szczygielski JJ, Bwanya PR, Charteris A, Brzeszczyński J (2021). The only certainty is uncertainty: an analysis of the impact of COVID-19 uncertainty on regional stock markets. Financ Res Lett.

[CR82] Topcu M, Gulal OS (2020). The impact of COVID-19 on emerging stock markets. Financ Res Lett.

[CR83] Vlastakis N, Markellos RN (2012). Information demand and stock market volatility. J Bank Financ.

[CR84] Wei X, Han L (2021). The impact of COVID-19 pandemic on transmission of monetary policy to financial markets. Int Rev Financ Anal.

[CR85] Wen F, Xu L, Ouyang G, Kou G (2019). Retail investor attention and stock price crash risk: evidence from China. Int Rev Financ Anal.

[CR86] Yousefinaghani S, Dara R, Mubareka S, Papadopoulos A, Sharif S (2021). An analysis of COVID-19 vaccine sentiments and opinions on Twitter. Int J Infect Dis.

[CR87] Zaremba A, Kizys R, Aharon DY, Demir E (2020). Infected markets: novel coronavirus, government interventions, and stock return volatility around the globe. Financ Res Lett.

[CR88] Zhang D, Hu M, Ji Q (2020). Financial markets under the global pandemic of COVID-19. Financ Res Lett.

